# Strategies for improving approximate Bayesian computation tests for synchronous diversification

**DOI:** 10.1186/s12862-017-1052-6

**Published:** 2017-08-24

**Authors:** Isaac Overcast, Justin C. Bagley, Michael J. Hickerson

**Affiliations:** 1 Biology Department, City College of New York, New York, NY 10031 USA; 2 The Graduate Center, City University of New York, New York, NY 10016 USA; 3 Departamento de Zoologia, Universidade de Brasília, Brasília, DF 70910-900 Brazil; 4 Departamento de Zoologia e Botânica, IBiLCE, Universidade Estadual Paulista, São José do Rio Preto, SP 15054-000 Brazil

**Keywords:** Approximate Bayesian computation (ABC), Comparative phylogeography, Divergence times, Synchronous diversification

## Abstract

**Background:**

Estimating the variability in isolation times across co-distributed taxon pairs that may have experienced the same allopatric isolating mechanism is a core goal of comparative phylogeography. The use of hierarchical Approximate Bayesian Computation (ABC) and coalescent models to infer temporal dynamics of lineage co-diversification has been a contentious topic in recent years. Key issues that remain unresolved include the choice of an appropriate prior on the number of co-divergence events (Ψ), as well as the optimal strategies for data summarization.

**Methods:**

Through simulation-based cross validation we explore the impact of the strategy for sorting summary statistics and the choice of prior on Ψ on the estimation of co-divergence variability. We also introduce a new setting (β) that can potentially improve estimation of Ψ by enforcing a minimal temporal difference between pulses of co-divergence. We apply this new method to three empirical datasets: one dataset each of co-distributed taxon pairs of Panamanian frogs and freshwater fishes, and a large set of Neotropical butterfly sister-taxon pairs.

**Results:**

We demonstrate that the choice of prior on Ψ has little impact on inference, but that sorting summary statistics yields substantially more reliable estimates of co-divergence variability despite violations of assumptions about exchangeability. We find the implementation of β improves estimation of Ψ, with improvement being most dramatic given larger numbers of taxon pairs. We find equivocal support for synchronous co-divergence for both of the Panamanian groups, but we find considerable support for asynchronous divergence among the Neotropical butterflies.

**Conclusions:**

Our simulation experiments demonstrate that using sorted summary statistics results in improved estimates of the variability in divergence times, whereas the choice of hyperprior on Ψ has negligible effect. Additionally, we demonstrate that estimating the number of pulses of co-divergence across co-distributed taxon-pairs is improved by applying a flexible buffering regime over divergence times. This improves the correlation between Ψ and the true variability in isolation times and allows for more meaningful interpretation of this hyperparameter. This will allow for more accurate identification of the number of temporally distinct pulses of co-divergence that generated the diversification pattern of a given regional assemblage of sister-taxon-pairs.

**Electronic supplementary material:**

The online version of this article (doi:10.1186/s12862-017-1052-6) contains supplementary material, which is available to authorized users.

## Background

Over the last three decades, comparative phylogeographic studies have used population-level genetic data from regional biotic assemblages to investigate how earth history dynamics have contributed to regional patterns of biodiversity and community assembly [1–5]. The implementation of hierarchical Approximate Bayesian Computation [6–9] has become a key statistical approach in comparative phylogeography for using coalescent models to test hypotheses of vicariance and dispersal [10], synchronous isolation [11], and models of co-expansion [12, 13] and has subsequently been adopted in a diversity of fields including neurobiology and astronomy [8, 9]. Other statistical approaches have been recently developed for the analysis of comparative phylogeography under unified multi-species models [14], and hABC models using random-forest classification has been recently expanded to accommodate reduced-genome SNP data [4, 14, 15]. However, hABC was initially developed for the purpose of quantifying patterns of co-isolation across taxon-pairs [12] based on datasets typically consisting of animal mtDNA sequences.

In recent years, there has been some disagreement [16–18] concerning two key details of how hABC is implemented in the MTML-msBayes software pipeline [7]. Leaving aside the disagreements about careful choice of the prior distribution of divergence times, Pr(τ), and its impact on the finite sampling of hyperparameter space when implementing hABC [17], two other contested issues are how best to sample from the prior on the number of co-divergence events (Ψ) and whether to sort the summary statistic vector prior to the rejection step. For the former, it has been argued that implementing a Dirichlet-process prior (DPP) over Ψ reduces bias that leads to incorrect inference of synchronous divergence given a discrete uniform prior over Ψ [19]. For the latter, an argument has been made that sorting the summary statistic vector by ascending values of average pairwise divergence (π_b_), in order to improve computational efficiency and accuracy in estimating the dispersion index of divergence times (Ω = Var(τ)/E(τ)), introduces bias favoring overestimation of the degree of synchronicity in divergence times [19]. Furthermore, it has been argued that using the sorting option leads to biased results and should be avoided because it treats species-specific elements within any one of the summary statistic classes as *exchangeable* across taxon-pair samples, irrespective of differences across taxa with regards to sequence length, number of sequences, DNA substitution model, as well as differences in mutation rates, and ploidies [19]. However, most of the summary statistic classes have equal expectation across numbers of individuals and sequence length (they are largely scaled per base pair) yet will have unequal variances. Furthermore, if there are differences in mutation rates, substitution models, or ploidies (which are all accommodated in the simulation step), this will be reflected consistently across simulations and the associated sorted vectors accordingly. While this sorting violates some of these aforementioned assumptions, it is likely that hABC estimation is robust to these deviations in most if not all single locus mtDNA datasets. In this case, violations of exchangability by sorting the vector are outweighed by information gained about the distribution of divergence times, similar to how the rank abundance curve extracts information about species abundance distributions in community ecology. As is common in models of population genetics, minor violations of some assumptions can lead to improved inference, and in this case the sorted vector can result in a greater potential correlation between the variability in divergence times and the distribution of summary statistic values within each summary statistic class of the sorted vector. Luckily one can always use simulation experiments to compare the efficacy of alternative summary statistic options by quantifying differences in accuracy and bias in posterior estimates given identical datasets simulated under known parameter values.

Beyond disagreements over these options, the far more fundamental factor underlying the difficulty in estimating the Ψ hyperparameter is the inherent lack of correlation between Ψ and the actual variability in divergence times. For example, if many distinct divergence events are temporally clustered in time, the correspondingly high Ψ value will have little relevance to the overall level of high synchronicity of divergences in a given assemblage. At the other extreme, Ψ = 2 could involve two co-divergence pulses that were 1 million years apart, corresponding to an overall high level of temporal discordance in the assemblage as manifested by high variability in pairwise divergences.

To improve upon estimation of Ψ, we introduce and demonstrate the implementation of a user-defined buffer, β, to enforce a minimum time between any two co-divergence pulses [4, 14, 15]. Appropriate values of β should increase the correlation between Ψ and the true variability in divergence times, thereby increasing the utility of Ψ for meaningful interpretation in the context of answering biogeographic questions at Pleistocene and Pliocene time scales. For example, if co-divergence events are hypothesized in the context of Quaternary glaciation cycles, one may be interested in testing whether a temperate community assemblage became significantly fractured during any one of the late Pleistocene glacial maxima or minima. In this case, one might consider using β values equivalent to 25,000 years such that divergences occurring within temporal windows of 50,000 years would be considered effectively part of the same co-divergence pulse.

In this paper, we use simulation-based cross-validation to evaluate how different β values impact the estimation of Ψ and Ω. We also implement a simulation-based two-by-two experiment to compare Ψ and Ω estimators under contrasting options for sorting the summary statistic vector, as well as contrasting the use of discrete uniform and DPP prior for Ψ. Finally, using what we learned from these simulation experiments, we reanalyze three sets of empirical data from assemblages representing a range of life-histories, data configurations, and sample sizes, such as are routinely analyzed in mitochondrial-based comparative phylogeographic studies.

## Methods

### Implementing buffered divergence times

Under the msBayes model, the number of co-divergence times is parameterized by Ψ under a discrete uniform prior. The value of Ψ can range from 1 to *Y*, where *Y* equals the number of taxon-pairs in question. A Ψ value of 1 signifies simultaneous divergence of all taxa, whereas Ψ = *Y* indicates fully asynchronous divergence. Given any history of more than one divergence time (Ψ > 1), any subset of the divergence times within the vector {τ_0_ *, …,* τ_Ψ_} can be tightly clustered in time, making this parameter difficult to meaningfully interpret with respect to understanding the distribution of co-divergence pulses. In other words, this original parameterization of Ψ results in a poor correlation with the actual variability in divergence times and the summary statistics underlying this variability (π_b_ and π_net_; [20]) and is a poor indicator for how many pulses of co-divergence there might actually have been. Although overall variability in τ is well quantified by Ω, the dispersion index of divergence times (Var(τ)/E(τ)), having good estimates of Ψ can be important for testing more detailed biogeographic hypotheses.

A more desirable property for Ψ would be for it to better reflect the effective number of divergence pulses. To this end, we implemented a modification to the MTML-msBayes [7] algorithm for sampling divergence times (*τ*) between pairs of sister taxa. As in the original implementation, 1 to Ψ different divergence times are randomly sampled from a prior distribution for τ. However, during the sampling process we incorporate a condition such that any two pulses of co-divergence within the vector {τ_0_ *, …,* τ_Ψ_} of divergence times are separated by at least β units of time, effectively generating a buffer around divergence times of width 2*β. The process of randomly drawing each of the divergence times from the prior given each hyperprior draw of Ψ continues until all taxon-pairs are assigned divergence times conditional that all Ψ times are at least β coalescent time units from each other. Due to the dynamic between β and the uniform prior for τ, the upper limit of the discrete uniform prior for Ψ may be truncated since this limit is determined by τ / 2β. For example, if τ_max_ = 0.75 and β is set to 0.1, τ_max_ / 2β = 3.75 and therefore the maximum possible value of Ψ_max_ will be 3.

### Simulation experiments assessing impact of β

We investigated the impact of varying β values on estimation of Ψ and Ω under a range of data configurations. To maintain direct comparison with previous simulation experiments of MTML-msBayes conducted by [21], we included a data configuration consistent with 18 sister-taxon-pairs of parasitoid wasps codistributed across the Iberian and Balkan regions. We also included two smaller data configurations of co-distributed Panamanian frogs (*n* = 4 taxon-pairs) and fishes (*n* = 3 taxon-pairs) (Bagley et al. in review). These data configurations reflect numbers of taxon-pairs, numbers of samples per taxon, and numbers of base pairs per locus commonly evaluated in single locus comparative phylogeographic studies [22–24]. In all three cases, we used simulation experiments to explore the bias and accuracy in estimates of Ψ and Ω given four different β values (0.0, 0.01, 0.05, and 0.1), using leave-one-out cross-validation [25]. For each β value and data configuration, we generated a reference table of 3 × 10^6^ random draws from the hyperprior. We then generated 100 PODS under the same β value and data configuration to compare the true values and mode estimates of Ψ and Ω. We quantified the impact of β on these estimates by calculating root mean squared error (RMSE) rates in aggregate, as well as RMSE as a function of the true parameter values.

### Simulation experiments assessing summary statistic sorting options and hyperprior on Ψ

It has been suggested that the default option of resorting the summary statistics within the vector **D** by the magnitude of π_b_, as described in [7], should be avoided for most empirical datasets [19] due to the possible lack of *exchangeability* of summary statistic classes across taxon-pairs potentially biasing towards the inference of synchronous co-divergence. Additionally, it has been suggested that the discrete uniform prior on Ψ also biases toward inference of synchronous co-divergence [19]. To directly compare the ability to estimate Ψ and Ω given these different decisions, we conducted a two-by-two experiment including both sorting strategy and the two hyperpriors for Ψ: the DPP as described by [19], and the discrete uniform hyperprior first introduced by [12]. We accomplished this by porting the implementation of the DPP from DPP-msbayes [19] into MTML-msBayes [7]. The new implementation along with all iPython notebook code for reproducing simulations and analyses are available on GitHub (https://github.com/Hickerlab/msBayes).

To increase comparability of the results for each hyperprior for Ψ, the sorted and unsorted reference tables of 3 × 10^6^ draws were generated from identically sampled random draws from the hyperprior with the only difference being the sorting strategy for **D**. This was done for all four combinations of sorting strategies and alternative hyperpriors for Ψ (DPP and uniform) to generate four different combinations of hABC options hereafter referred to as sorted uniform (U_S_), sorted DPP (DPP_S_), unsorted uniform (U_U_), and unsorted DPP (DPP_U_). For each estimate given an associated POD, we retained 1000 posterior samples for each hABC posterior estimate under each of the four reference tables associated with the four different hABC settings (i.e. U_S_, DPP_S_, U_U_, and DPP_U_). We used non-linear neural network regression during the post-acceptance rejection adjustment for Ψ [26], and local linear regression for Ω [27]. For this simulation experiment, we used the same three data configurations as above (3, 4, and 18 taxon-pairs). As above, we quantified error by calculating aggregate RMSE, as well as RMSE as a function of the true parameter values.

### Applications to empirical data: Neotropical butterflies, frogs, and fishes

To explore the effects of various magnitudes of β on estimation of Ψ and Ω for real data, we reanalyzed three empirical datasets. This included data from Bagley et al. (in review) that tested variation in diversification times among population-pairs of Panamanian frogs (*n* = 4 taxon-pairs) and freshwater fishes (*n* = 3 taxon-pairs) using one mitochondrial sequence from each of 10 to 28 individuals per taxon. We also reanalyzed a large dataset of 116 sister species-pairs of Neotropical butterflies consisting of an average of 3.4 mitochondrial cytochrome c oxidase subunit I sequences per taxon [28]. Guided by our simulation investigation, we adopted a uniform prior on Ψ with order-independent sorting of the summary statistic vector and report results based on β = 0.0, 0.05, 0.01, and 0.1 (See Additional file 1 for complete details of empirical analysis methods).

To test the goodness of fit of our model, we used prior and posterior predictive simulations [29] implemented in the *abc* R package. For the the prior predictive goodness of fit, we plot the first two principal components of the observed and accepted simulated summary statistics given the approximate posterior distribution. For the posterior predictive goodness of fit test, we tested whether the observed summary statistics fell within the distribution of summary statistics that were calculated from re-simulated data given the approximate posterior distribution of Ψ.

## Results

### Impact of β on estimation of Ψ and Ω

**Fig. 1:**
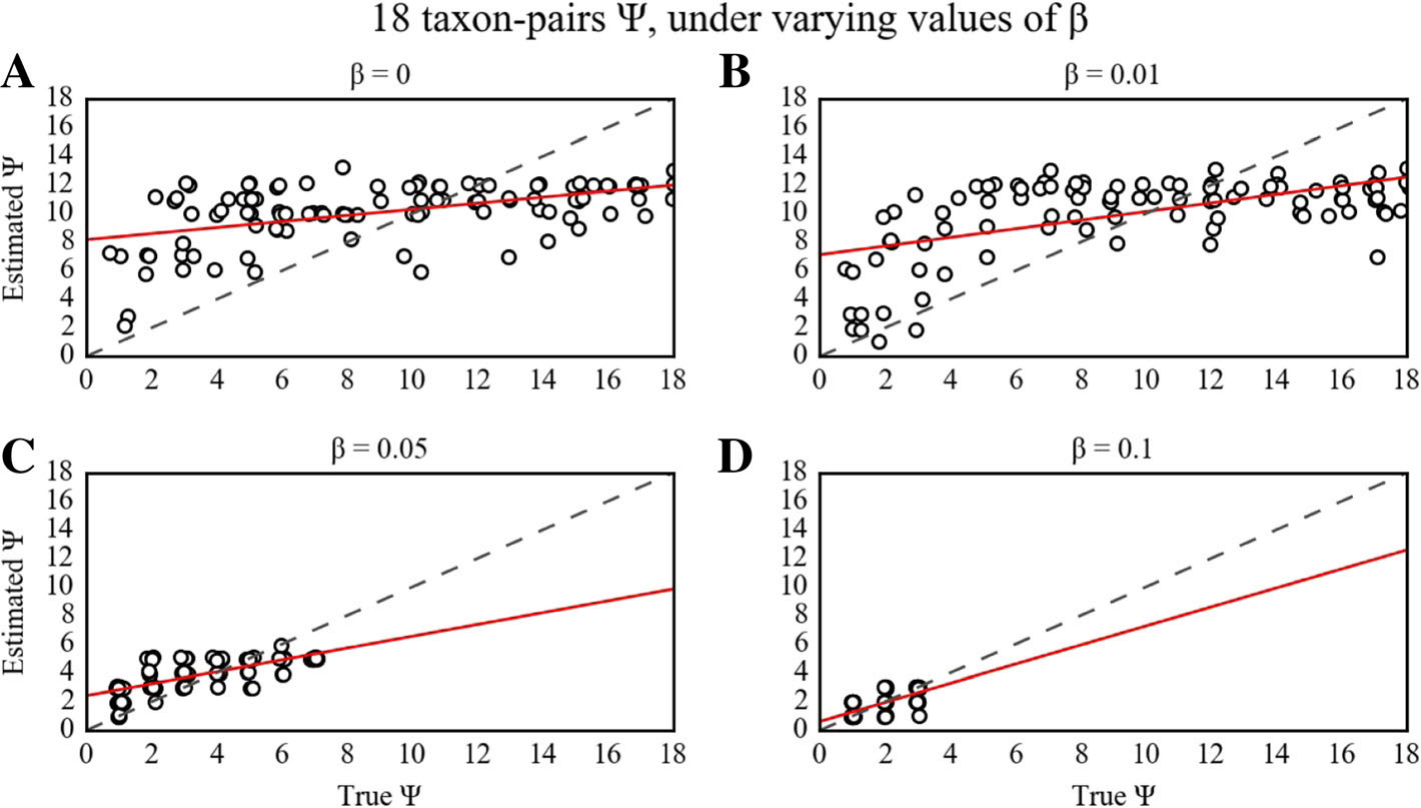
PODS results for estimation of Ψ under varying values of β for the 18 taxon-pair configuration. Scatterplots of true versus estimated values of Ψ for 100 PODS across different buffering regimes for the 18 taxon-pair data configuration. PODS were simulated and analyzed with a uniform prior on Ψ and sorted summary statistics using reference tables composed of 3 × 10^6^ samples from the prior. Points in the plot are slightly perturbed to visualize the number of points for each estimate. The dashed line is the identity line, and the red line is a simple linear regression of estimated Ψ onto true Ψ. **a** β = 0 **b** β = 0.01 **c** β = 0.05 **d** β = 0.1

**Fig. 2:**
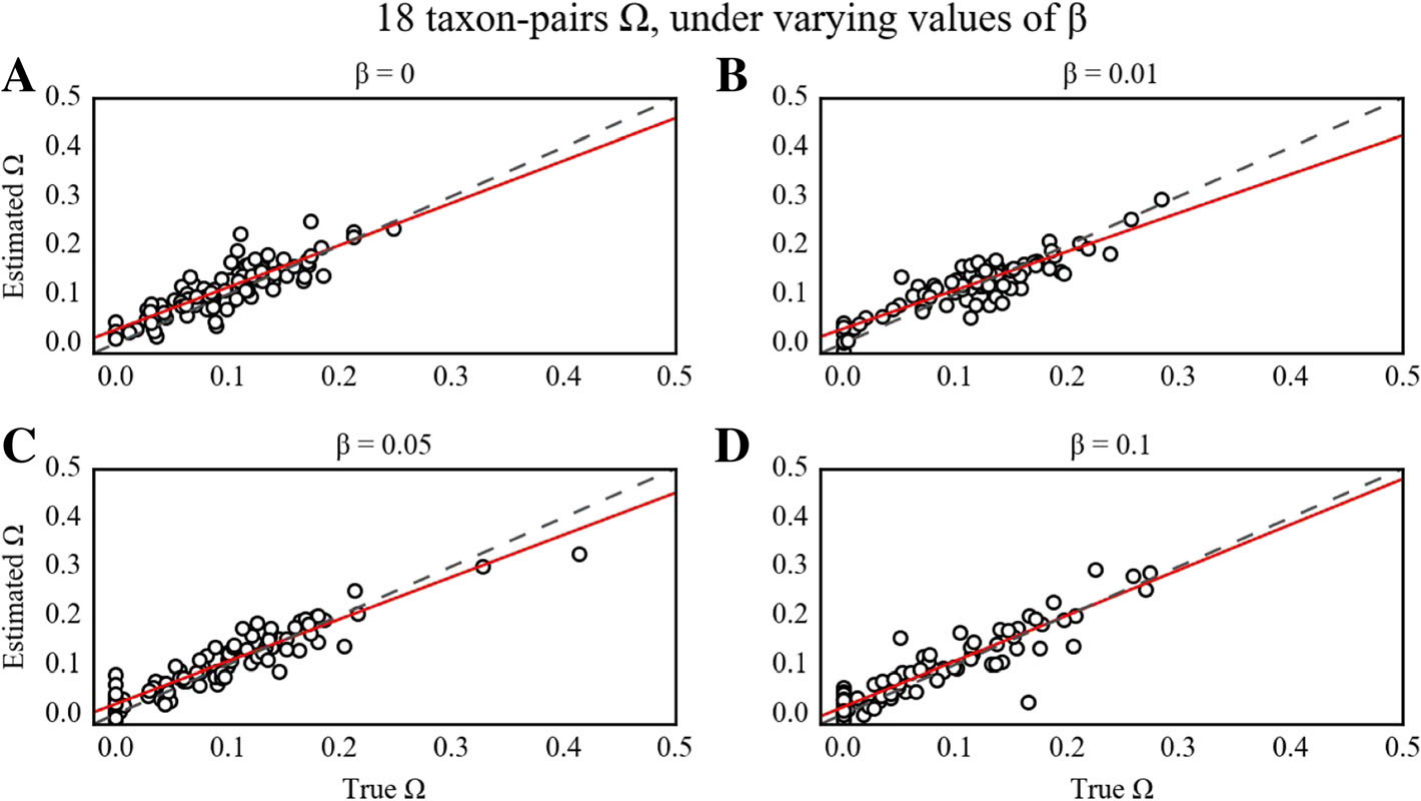
PODS results for estimation of Ω under varying values of β for the 18 taxon-pair configuration. Scatterplots of true versus estimated values of Ω for 100 PODS across different buffering regimes for the 18 taxon-pair data configuration. PODS were simulated and analyzed with a uniform prior on Ψ and sorted summary statistics using reference tables composed of 3 × 10^6^ samples from the prior. The dashed line is the identity line, and the red line is a simple linear regression of estimated Ω onto true Ω. **a** β = 0 **b** β = 0.01 **c** β = 0.05 **d** β = 0.1

For the 18-taxon-pair configuration, implementation of the β threshold generally resulted in substantial improvements for estimating Ψ, while improvements on Ω estimates were more conditional on true values of Ω. The highest accuracy in estimating Ψ was achieved with β = 0.05 or 0.1, whereas a β = 0.01 resulted in negligible improvement in estimation over β = 0 (Table 1; Figs. 1 and 2). In contrast to the 18-taxon case, there was little improvement in the estimation of Ψ given the smaller 3 and 4 taxon-pair datasets across all the values of β that we explored (Table 1; Additional file 1: Figs. S9, S10, S11, S12, S13, S14, S15 and S16). Although overall estimates of Ω did not markedly improve with increasing β for the 3 or 4 taxon-pair cases, the probability of incorrectly inferring synchronous divergence (Ω < 0.01) was lowest with the highest value of β modeled (β = 0.1).

**Table 1:** RMSE on estimates of Ψ and Ω sampling the prior and PODS with varying β

β	18 taxon-pairs U_S_ Ψ	18 taxon-pairs U_S_ Ω	18 taxon-pairs U_U_ Ψ	18 taxon-pairs U_U_ Ω	4 taxon-pairs U_S_ Ψ	4 taxon-pairs U_S_ Ω	3 taxon-pairs U_S_ Ψ	3 taxon-pairs U_S_ Ω
0	4.443	0.0314	5.0695	0.064	0.866	0.1347	0.7874	0.0895
0.01	4.4531	0.0273	5.2278	0.0761	0.911	0.102	0.8062	0.0959
0.05	1.5264	0.0276	1.8055	0.0507	0.911	0.146	0.8	0.1048
0.1	0.6164	0.0298	0.8646	0.0392	0.9747	0.1317	0.7681	0.0909

Mean RMSE in estimation of Ψ and Ω averaged across 100 PODS for each of the three data configurations under varying β values. These experiments were conducted with a uniform prior on Ψ, sorted summary statistics vectors, and 3 × 10^6^ samples from the prior distribution

### Impact of summary statistic ranking and hyperprior of Ψ on the estimation of Ω and Ψ

The summary statistics sorting strategy had considerable effects on estimating Ω, while it had lesser impact on estimating Ψ, dependent on the size of the dataset. This dynamic was most apparent for the larger dataset of 18 taxon-pairs, for which RMSE scores were 3-fold lower for the estimation of Ω when using the sorting algorithm (U_S_ and DPP_S_), regardless of prior choice for Ψ (Table 2). This result contrasts sharply with previously published studies that advocate using an unsorted summary statistic vector and DPP hyperprior for Ψ [19, 30]. Not only does sorting improve estimation of Ω markedly, but we also found that it had some impact on improving the estimation of Ψ given larger datasets.

**Table 2:** RMSE on Ψ and Ω varying the prior on Ψ and the sorting method

	18 taxon-pairs Ψ	18 taxon-pairs Ω	4 taxon-pairs Ψ	4 taxon-pairs Ω	3 taxon-pairs Ψ	3 taxon-pairs Ω
U_S_	4.6079; **4.141**	0.0298; **0.0295**	0.9695; **0.974**	0.1052; **0.0952**	0.8000; **0.8069**	0.0850; **0.087**
U_U_	5.1069; **4.892**	0.0867; **0.0856**	0.9592; **1.038**	0.1242; **0.1139**	0.8367; **0.7625**	0.0949; **0.091**
DPP_S_	5.339; **5.317**	0.0286; **0.0303**	1.0149; **0.698**	0.1158; **0.1073**	0.8246; **0.7937**	0.0720; **0.0845**
DPP_U_	5.8853; **5.058**	0.0868; **0.0854**	0.9; **0.92**	0.1262; **0.1245**	0.8124; **0.7454**	0.0954; **0.092**

Mean RMSE in estimation of Ψ and Ω averaged across 100 PODS for each of the three data configurations under each of the four models we tested. In the baseline text of each model “U” and “DPP” refer to the two priors on Ψ that were tested; the uniform distribution and the Dirichlet-process prior, respectively. In the subscript text “S” and “U” refer the sorting method applied; sorted and unsorted summary statistics vectors, respectively. The first value in each cell was calculated from reference tables composed of 3 × 10^6^ samples from the prior. The second value in each cell was calculated using 5 × 10^7^ samples from the prior (in bold)

**Fig. 3:**
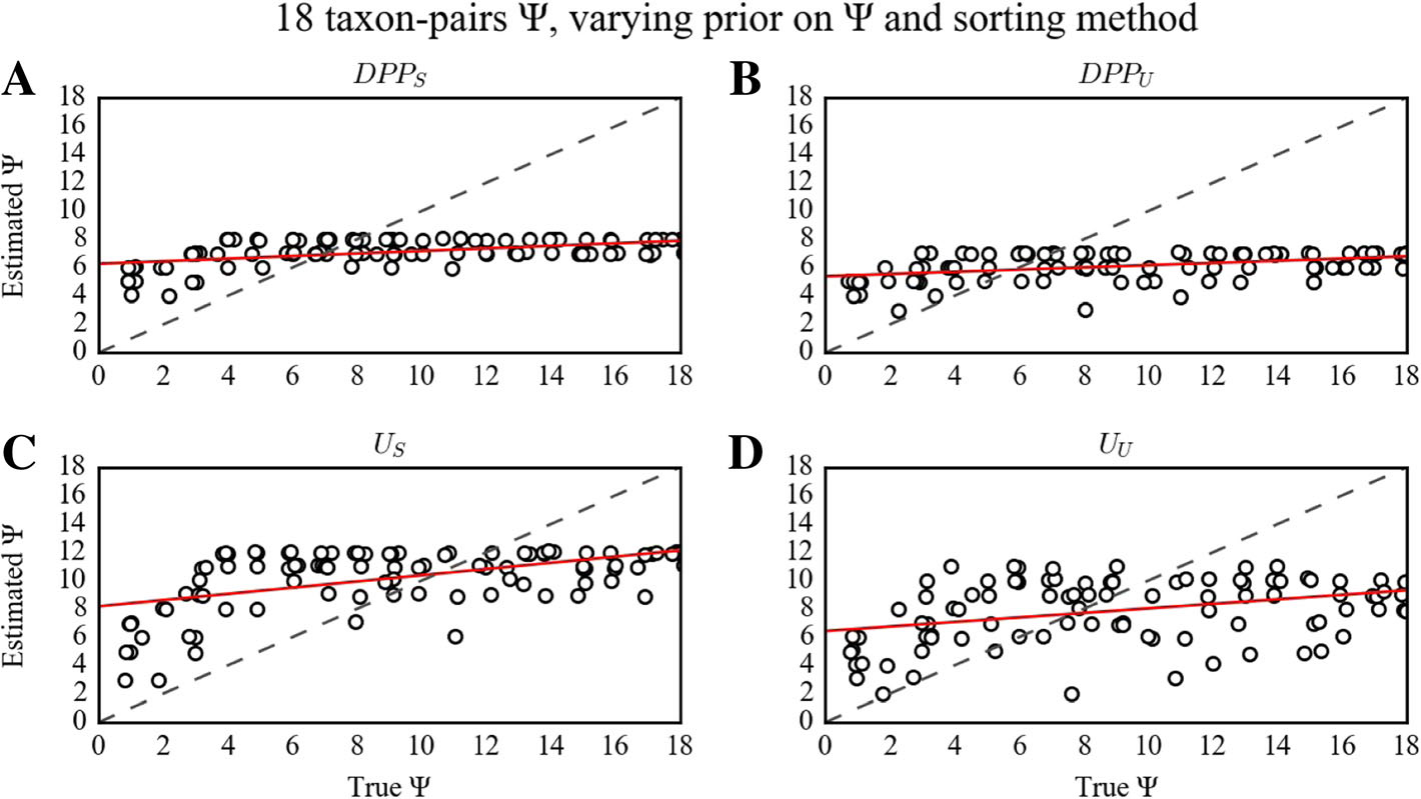
PODS results for estimation of Ψ under varying prior on Ψ and sorting method for the 18 taxon-pair configuration. Scatterplots of true versus estimated values of Ψ for 100 PODS under different models of prior distribution on Ψ and applying different sorting strategies for the 18 taxon-pair data configuration. PODS were analyzed using reference tables composed of 3 × 10^6^ samples from the prior. Points in the plot are slightly perturbed to visualize the number of points for each estimate. The dashed line is the identity line, and the red line is a simple linear regression of estimated Ψ onto true Ψ. **a**) Dirichlet-process prior with sorted summary statistics. **b**) Dirichlet-process prior with unsorted summary statistics. **c**) Uniform prior with sorted summary statistics. **d**) Uniform prior with unsorted summary statistics

**Fig. 4:**
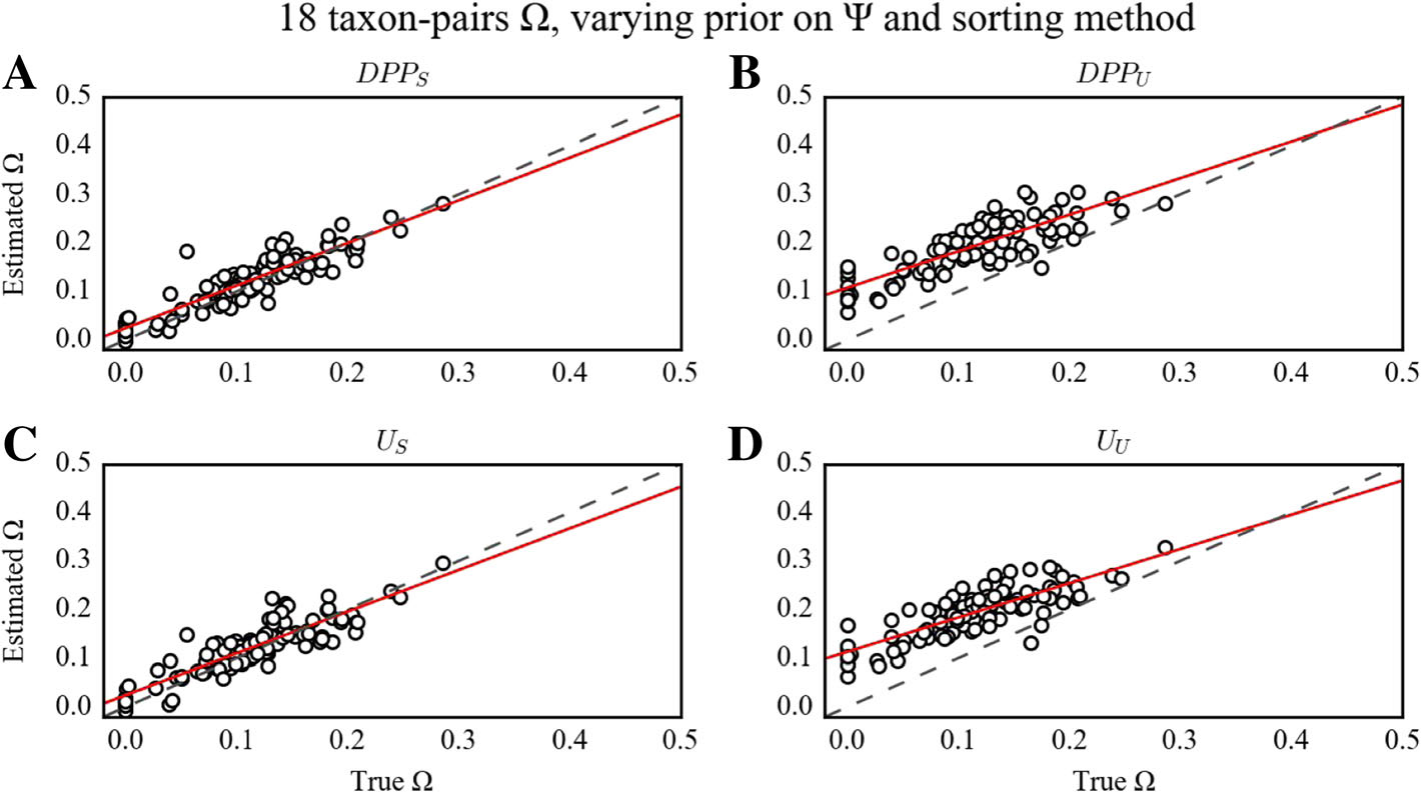
PODS results for estimation of Ω under varying prior on Ψ and sorting method for the 18 taxon-pair configuration. Scatterplots of true versus estimated values of Ω for 100 PODS under different models of prior distribution on Ψ and applying different sorting strategies for the 18 taxon-pair data configuration. PODS were analyzed using reference tables composed of 3 × 10^6^ samples from the prior. The dashed line is the identity line, and the red line is a simple linear regression of estimated Ω onto true Ω. **a**) Dirichlet-process prior with sorted summary statistics. **b**) Dirichlet-process prior with unsorted summary statistics. **c**) Uniform prior with sorted summary statistics. **d**) Uniform prior with unsorted summary statistics

The effects of the two alternative hyperpriors on Ψ have only minor effects on posterior estimates of Ψ and Ω. Across the three taxon-sample sizes (*n* = 3, 4, and 18 taxon-pairs) and sorting algorithms, choice of the hyperprior on Ψ did not strongly impact estimation of Ψ (Fig. 3, Additional file 1: Figs. S1 and S2) or Ω (Fig. 4, Additional file 1: Figs. S3 and S4). However, using the DPP (DPP_S_ and DPP_U_) hyperprior consistently resulted in slightly more accurate estimates of Ψ given the smaller 3 and 4 taxon-pair cases, whereas given the 18 taxon-pair case, the uniform prior on Ψ (U_S_ and U_U_) resulted in slightly improved estimates of Ψ (Table 2).

**Fig. 5:**
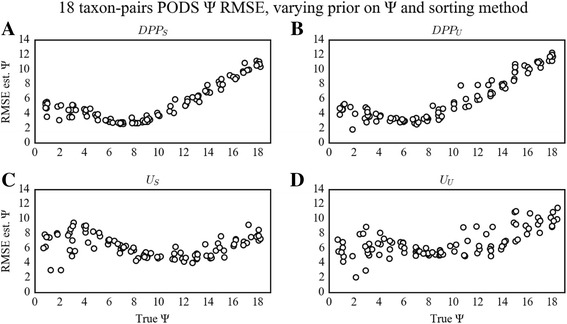
PODS results for RMSE on estimation of Ψ under varying prior on Ψ and sorting method for the 18 taxon-pair configuration. Scatterplots of RMSE in estimation of Ψ as a function of true Ψ for 100 PODS under different models of prior distribution on Ψ and applying different sorting strategies for the 18 taxon-pair data configuration. PODS were analyzed using reference tables composed of 3 × 10^6^ samples from the prior. Points in the plot are slightly perturbed to visualize the number of points for each estimate. **a**) Dirichlet-process prior with sorted summary statistics. **b**) Dirichlet-process prior with unsorted summary statistics. **c**) Uniform prior with sorted summary statistics. **d**) Uniform prior with unsorted summary statistics

**Fig. 6:**
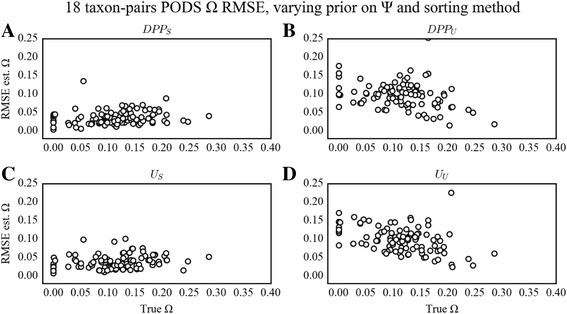
PODS results for RMSE on estimation of Ω under varying prior on Ψ and sorting method for the 18 taxon-pair configuration. Scatterplots of RMSE in estimation of Ω as a function of true Ω for 100 PODS under different models of prior distribution on Ψ and applying different sorting strategies for the 18 taxon-pair data configuration. PODS were analyzed using reference tables composed of 3 × 10^6^ samples from the prior. **a**) Dirichlet-process prior with sorted summary statistics. **b**) Dirichlet-process prior with unsorted summary statistics. **c**) Uniform prior with sorted summary statistics. **d**) Uniform prior with unsorted summary statistics

However, the effects of the chosen hyperprior on Ψ were dependent upon the true Ψ and Ω values across all taxon-pair configurations. Given the 18 taxon-pair configuration and the DPP hyperprior on Ψ (DPP_S_ or DPP_U_), we found a concave pattern of error probabilities quantified by RMSE, with the lowest error at true Ψ = 7 regardless of sorting options. This yielded moderately increasing error rates as Ψ approached 1 and 18, with the highest error rates occurring when true Ψ > 12 (Fig. 5). Given the 3 and 4 taxon-pair configurations and the DPP hyperprior on Ψ (DPP_S_ or DPP_U_), the RMSE decreased as true Ψ increased regardless of sorting method. Unlike the error in estimation of Ψ, the RMSE on the estimation of Ω was dependent on sorting method and true Ω value (Fig. 6). For the 18 taxon-pair configuration and DPP_S_, the RMSE was low and unbiased given true Ω. However, with DPP_U_, RMSE was generally higher and demonstrated an upward bias for decreasing values of true Ω. This bias was also present in the 3 and 4 taxon-pair configurations, but was less pronounced (Additional file 1: Figs. S5, S6, S7 and S8).

Unlike the error dynamics associated with DPP_S_ and DPP_U_, use of U_S_ and U_U_, under all three data configurations (3, 4, and 18 taxon-pairs) resulted in estimation error that was more evenly distributed across all simulated true Ψ values regardless of sorting method (Fig. 3). Likewise, the RMSE on the estimation of Ω was generally low and unbiased across all true Ω values when using the sorting method (U_S_). On the other hand, with U_U_ there was an increasing bias in RMSE with decreasing true values of Ω (Fig. 4). This pattern was similar across all data configurations, but was most pronounced with the 18 taxon-pair configuration.

### Application to empirical datasets

For both the frogs (*n* = 4 taxon-pairs) and fishes (*n* = 3 taxon-pairs), using a β value of 0.05 resulted in equivocal inference of simultaneous divergence across both datasets given both estimates of Ω and Ψ (Bayes factor 1.80 and 1.41 for the frogs and fishes, respectively; Table 3). Our simulation experiments suggested that the use of β does not appreciably change the estimation of co-divergence given a small number of taxon-pairs, and as such, it is unsurprising that dropping this setting also resulted in a similar inference of simultaneous divergence with BF(Ψ = 1, Ψ > 1 | β = 0) = 1.14 for the frogs and BF(Ψ = 1, Ψ > 1 | β = 0) = 1.33 for the fishes (Table 3).

**Table 3:** Mode estimates of Ψ and Ω for empirical datasets under the most likely model, with and without buffering

	β	β_years_	Ψ_mode_	E[*τ*]	Ω_mode_	BF(Ψ = 1,Ψ > 1)
Frogs	0	N/A	1	0.106	0.0036	1
	0.05	8.0 × 10^4^	1	0.486	0.010	1.80
Fishes	0	N/A	1	0.054	0.0017	1
	0.05	8.9 × 10^4^	1	0.198	0.003	1.41
Butterfly (Full)	0	N/A	15	0.378	0.108	~0
	0.05	3.2 × 10^4^	5	0.389	0.113	~0

Mode estimates for Ψ and Ω, and mean and variance of *τ* estimated for each of the empirical datasets (three fish, four frog, three butterfly and 116 butterfly taxon-pairs). β, E[τ], and Ω_mode_ are reported in coalescent units. Β_years_ is size of the buffer rescaled to years based on the assumed mutation rates and generation times of the focal taxa. All empirical analyses were performed with a uniform prior on Ψ, sorted summary statistics vectors, and reference tables composed of 3 × 10^6^ samples from the prior distribution

For the reanalyisis of the 116 taxon-pairs of Neotropical butterflies, estimates of Ω and Ψ both suggest that asynchronous divergence was prevalent within butterflies across the Neotropics and, as predicted, the implementation of β resulted in an estimate of fewer co-divergence pulses than when β was set to 0. Similar to the smaller datasets, the mode and 95% HPD of Ω were not significantly different across buffering regimes (Mode estimate of Ω: β(0) = 0.11; β(0.01) = 0.12; β(0.05) = 0.11; β(0.1) = 0.12). However, increasing values of β had a strong impact on estimates of Ψ. Though (Pr(Ψ > 1) ≈ 1) held true across all buffering regimes, mode estimates varied considerably (Mode estimate of Ψ: β(0) = 15; β(0.01) = 24; β(0.05) = 5; β(0.1) = 5). This finding is in agreement with the divergence time estimates of Garzón-Orduña et al. [25], who obtained a wide range of divergence times without accounting for differences in population divergences and gene divergence predicted under the standard coalescent.

Goodness-of-fit tests based on the approximate posterior distribution for all three empirical datasets showed that, for all β values, the observed data fell within the cloud of simulated points in the PC plots (Additional file 1: Figs. S17, S18 and S19). Similarly, results of our posterior predictive simulations indicated that the observed summary statistics were not significantly different from the simulated summary statistics (*p* > 0.05 for all values of β), indicating a good fit of our model. Although the invariance of divergence that is assumed within each of the Ψ divergence pulses in our model using β is a simplified approximation, the prior and posterior predictive checks we implemented both show an acceptable goodness of fit demonstrating that our inferential model can generate all four datasets, a fundamental step for Approximate Bayesian Computation [29, 31, 32].

## Discussion

### Buffering improves estimation of co-divergence events

The dynamics between chosen β and *τ*_max_ values are important, as these constrain the sampling space of *τ* as well as the maximum value of Ψ, yet these dynamics also depend on the number of taxon-pairs analyzed. With few taxon-pairs, even relatively high values of β may not reduce the maximum value of Ψ (Ψ_max_). However, as the number of taxon-pairs increases, increasing the value of β will reduce Ψ_max_ proportional to the number of taxon-pairs (*Y*) and τ_max_. For example, the β values that we explored had little impact on Ψ_max_ for the 3 and 4 taxon-pairs data configurations. However, in the 18 taxon-pairs case, increasing values of β proportionally decreased Ψ_max_ (β = 0.05, Ψ_max_ = 7; β = 0.1, Ψ_max_ = 3). It is important to note that if τ_max_ / β < *Y* then Ψ_max_ < *Y*, which precludes the possibility of inferring fully idiosyncratic divergence. We recommend that researchers carefully consider β values that reflect a meaningful time scale of biogeographic interest in the context of co-demographic histories, and likewise consider *τ*_max_ values that are plausible with respect to observed gene-tree divergence times [17]. Obviously, choosing β = *τ*_max_ / 2 would not yield meaningful results, whereas a β value corresponding to 10,000–50,000 years would be within the level of resolution of interest for many biogeographic questions. Additionally, as with all comparative phylogeographic analysis, care must be taken in properly scaling time across study organism to account for variation in mutation rates, generation times and/or ploidy. The msBayes pipeline includes such a scaling factor per taxon per locus, to ensure meaningful inference of co-divergence within any given buffering regime.

An important simplification of our β implementation is that co-divergence times within a pulse are identical. While this is admittedly unrealistic, it is best to view adding a chosen β value to the model as a useful approximation that captures the overall pattern in the number of meaningful co-divergence pulses. One possible improvement to explore would be to allow each taxon-pair assigned to a particular *τ*, given a chosen β, to draw its own *τ* value from a normal distribution centered around *τ* with a standard deviation of 2β. This would maintain the integrity of the buffer as these taxon-pairs could still be considered to be “co-diverging”, but would also allow divergence times for each taxon-pair to vary in a way that could capture differences in timing due to stochastic and/or ecologically deterministic factors. A related complementary method could be developed by using the Poisson distribution to construct a test for overdispersion or clumpiness, with the null being a random distribution of divergence events across the prior on divergence times [33].

### Impact of hyperprior for Ψ

Although the motivation for the DPP is to allow researchers to distribute prior probability across divergence models conditional on the number of possible models per Ψ value [19], thereby reducing bias against inference of multiple co-divergence events, we find choice of the prior has little impact on the estimation of Ψ. Here we show that, given a finite and reasonable number of samples from the prior, the concave shape of the plot of RMSE estimates on Ψ across PODS for the DPP does reflect the preponderance of weight placed on the prior for values of Ψ(Fig. 3). By contrast the RMSE of the uniform estimates are more evenly distributed, reflecting the reduced constraint and the increased ability of the uniform prior to accurately infer a greater range of true Ψ values. While these sampling dynamics make the DPP less prone to incorrectly reject truly asynchronous divergence histories if basing such tests on the hyperparameter Ψ [19], this comes with the cost of reducing power to infer histories of true synchronous divergence. Moreover, other factors explored here and elsewhere clearly demonstrate Ω to be a far superior metric for this test if one implements the sorting option [17, 21].

### Application to empirical data

The effects of the choice of hyperprior for Ψ, implementation of β, and sorting of summary statistic elements were all reduced in the smaller datasets of 3 and 4 taxon-pairs, and as predicted, they did not change the outcome for the corresponding empirical frog and fish datasets. Inference from the larger butterfly dataset of 116 taxon-pairs was somewhat sensitive to these choices. Our estimate of Ω = 0.113 is consistent with an estimate of Ψ = 5 co-divergence pulses given β = 0.05. The prior and posterior predictive checks we implemented both show an acceptable goodness of fit, demonstrating that our inferential model can generate all three datasets, a fundamental step for hABC analysis [29, 33].

## Conclusions

We have demonstrated that estimating the number of pulses of co-divergence across co-distributed taxon-pairs is improved by applying a flexible buffering regime over divergence times. This achieves better statistical properties by increasing correlation between the number of co-divergence pulses and the overall magnitude of variability in divergence times. This will allow for more accurate identification of the number of temporally distinct pulses of co-divergence that generated the diversification pattern of a given regional assemblage of sister-taxon-pairs. Additionally, we show that the choice of hyperprior on Ψ is not as important as the choice of whether to sort the summary statistics vector, as our simulation experiments clearly show that using the sorting strategy results in drastically improved estimates of the overall variability in divergence. Although the assumptions of exchangeability across units within this vector may not be met for many mtDNA datasets, as with many cases in population genetics and phylogenetics, violation of such assumptions can be outweighed by the improved accuracy gained from using a model with reduced complexity. We find the differences in bias and accuracy across all parameterizations become negligible given datasets composed of smaller numbers of taxon-pairs. Further, we find summary statistics are more tightly correlated with parameters of interest for larger numbers of taxon-pairs. An analysis specifically targeting this question would illuminate this dynamic, yet in general it is expected that estimation of variability in parameter values will improve with increasing numbers of taxa, as demonstrated in [14]As with any application of ABC in population genetics and phylogeography [34], we recommend the standard practice of using simulation experiments to test for robustness, and the use of goodness-of-fit tests to explore various summary statistic options, settings, and model hyperpriors in the context of one’s data and the computational resources at hand [35, 36].

## Additional file

Additional file 1: Supplementary information. File contains Supporting Materials and Methods, Supplementary Tables S1 & S2, and Supplementary figs. S1–S20. (DOCX 2273 kb)

## References

[1] Avise JC, Arnold J, Ball RM, Bermingham E, Lamb T, Neigel JE (1987). Intraspecific phylogeography: the mitochondrial DNA bridge between population genetics and systematics. Annu Rev Ecol Syst.

[2] Arbogast BS, Kenagy GJ (2001). Comparative phylogeography as an integrative approach to historical biogeography. J Biogeogr.

[3] Hickerson MJ, Carstens BC, Cavender-Bares J (2010). Phylogeography’s past, present, and future: 10 years after Avise, 2000. Mol Phylogenet Evol.

[4] Prates I, Xue AT, Brown JL, Alvarado-Serrano DF, Rodrigues MT, Hickerson MJ (2016). Inferring responses to climate dynamics from historical demography in neotropical forest lizards. Proc Natl Acad Sci.

[5] Shaw KL, Gillespie RG (2016). Comparative phylogeography of oceanic archipelagos: hotspots for inferences of evolutionary process. Proc Natl Acad Sci.

[6] Hickerson MJ, Stahl E, Takebayashi N (2007). msBayes: pipeline for testing comparative phylogeographic histories using hierarchical approximate Bayesian computation. BMC Bioinformatics.

[7] Huang W, Takebayashi N, Qi Y, Hickerson MJ (2011). MTML-msBayes: approximate Bayesian comparative phylogeographic inference from multiple taxa and multiple loci with rate heterogeneity. BMC Bioinformatics.

[8] Turner BM, Van Zandt T (2014). Hierarchical approximate Bayesian computation. Psychometrika.

[9] Ishida EEO, Vitenti SDP, Penna-Lima M, Cisewski J, de Souza RS, Trindade AMM (2015). Cosmoabc: likelihood-free inference via population Monte Carlo approximate Bayesian computation. Astronomy Comput.

[10] Smith BT, McCormack JE, Cuervo AM, Hickerson MJ, Aleixo A, Cadena CD (2014). The drivers of tropical speciation. Nature.

[11] Hickerson MJ, Stahl EA, Lessios HA (2006). Test for simultaneous divergence using approximate Bayesian computation. Evolution.

[12] Chan YL, Schanzenbach D, Hickerson MJ (2014). Detecting concerted demographic response across community assemblages using hierarchical approximate Bayesian computation. Mol Biol Evol.

[13] Burbrink FT, Chan YL, Myers EA, Ruane S, Smith BT, Hickerson MJ (2016). Asynchronous demographic responses to Pleistocene climate change in eastern Nearctic vertebrates. Ecol Lett.

[14] Xue AT, Hickerson MJ (2015). The aggregate site frequency spectrum for comparative population genomic inference. Mol Ecol.

[15] Xue AT, Hickerson MJ. Multi-DICE: R package for comparative population genomic inference under hierarchical co-demographic models of independent single-population size changes. Mol Ecol Resour. 2017; doi:10.1111/1755-0998.12686/full. 10.1111/1755-0998.12686 PMC5724483 28449263

[16] Oaks JR, Sukumaran J, Esselstyn JA, Linkem CW, Siler CD, Holder MT (2013). Evidence for climate-driven diversification? A caution for interpreting ABC inferences of simultaneous historical events. Evolution.

[17] Hickerson MJ, Stone GN, Lohse K, Demos TC, Xie X, Landerer C (2014). Recommendations for using msBayes to incorporate uncertainty in selecting an abc model prior: a response to oaks et al. Evolution.

[18] Oaks JR, Linkem CW, Sukumaran J (2014). Implications of uniformly distributed, empirically informed priors for phylogeographical model selection: a reply to Hickerson et al. Evolution.

[19] Oaks JR (2014). An improved approximate-Bayesian model-choice method for estimating shared evolutionary history. BMC Evol Biol.

[20] Nei M, Li WH (1979). Mathematical model for studying genetic variation in terms of restriction endonucleases. Proc Natl Acad Sci.

[21] Stone GN, Lohse K, Nicholls JA, Fuentes-Utrilla P, Sinclair F, Schönrogge K (2012). Reconstructing community assembly in time and space reveals enemy escape in a western Palearctic insect community. Curr Biol.

[22] Marske KA, Leschen RAB, Buckley TR (2012). Concerted versus independent evolution and the search for multiple refugia: comparative phylogeography of four forest beetles. Evolution.

[23] Bagley JC, Johnson JB (2014). Phylogeography and biogeography of the lower central American Neotropics: diversification between two continents and between two seas. Biol Rev Camb Philos Soc.

[24] Satler JD, Carstens BC. Phylogeographic concordance factors quantify phylogeographic congruence among co-distributed species in the Sarracenia Alata pitcher plant system. Evolution. 2016; doi:10.1111/evo.12924. 10.1111/evo.12924 27076412

[25] Bertorelle G, Benazzo A, Mona S (2010). ABC as a flexible framework to estimate demography over space and time: some cons, many pros. Mol Ecol.

[26] Beaumont MA, Zhang W, Balding DJ (2002). Approximate Bayesian computation in population genetics. Genetics.

[27] Csilléry K, François O, Blum MGB (2012). Abc: an R package for approximate Bayesian computation (ABC). Methods ecol. Evolution.

[28] Garzón-Orduña IJ, Benetti-Longhini JE, Brower AVZ. Timing the diversification of the Amazonian biota: butterfly divergences are consistent with Pleistocene refugia. J Biogeogr 2014. doi:10.1111/jbi.12330/full.

[29] Lemaire L, Jay F, Lee I-H, Csilléry K, Blum MGB. Goodness-of-fit statistics for approximate Bayesian computation. arXiv preprint. 2016. arXiv:1601.04096.

[30] Papadopoulou A, Knowles LL (2016). Toward a paradigm shift in comparative phylogeography driven by trait-based hypotheses. Proc Natl Acad Sci.

[31] Gelman A, Carlin JB, Stern HS, Dunson DB, Vehtari A, Rubin DB. Bayesian data analysis. Boca Raton, FL: CRC press; 2014.

[32] Gruenstaeudl M, Reid NM, Wheeler GL, Carstens BC (2016). Posterior predictive checks of coalescent models: P2C2M, an R package. Mol Ecol Resour.

[33] Raup DM, Sepkoski JJ Jr. Mass extinctions in the marine fossil record. Science. 1982;215:1501–3. 10.1126/science.215.4539.1501 17788674

[34] Beaumont MA. Approximate Bayesian computation in evolution and ecology. Annu Rev Ecol Evol Syst 2010. doi:10.1146/annurev-ecolsys-102209-144621.

[35] Lintusaari J, Gutmann MU, Dutta R, Kaski S, Corander J (2017). Fundamentals and recent developments in approximate Bayesian computation. Syst Biol.

[36] Sunnåker M, Busetto AG, Numminen E, Corander J, Foll M, Dessimoz C (2013). Approximate Bayesian computation. Plos Comput Biol.

