# Supplementary material for: Strategies for improving approximate Bayesian computation tests for synchronous diversification
Source: BMC Evol Biol. 2017 Aug 24;17:203. doi: 10.1186/s12862-017-1052-6 (PMC5571621; doi:10.1186/s12862-017-1052-6)
Supplement: Additional file 1: — Supplementary information. File contains Supporting Materials and Methods, Supplementary Tables S1 & S2, and Supplementary figs. S1–S20. (DOCX 2273 kb) [file 12862_2017_1052_MOESM1_ESM.docx]

**Supporting Information**

**Strategies for improving approximate Bayesian computation tests for synchronous diversification**

**Supporting Materials and Methods
Application to empirical data: Panamanian Frogs and Fishes** Over the last 20 years, the Central American Isthmus has emerged as an important system for phylogeographic research [[1]](https://paperpile.com/c/Z0W1m7/KOoQ9). As a demonstration of our new time buffering implementation, we applied the msBayes buffering method to re-analyze data from a recent paper by Bagley et al. (in review) that tests for a synchronous pulse of diversification, versus multiple pulses of diversification, among seven population-pairs of Panamanian frogs and freshwater fishes. This dataset reflects a large portion of a growing number of taxa known to exhibit a shared phylogeographic break across the Pacific coast of Panama, with distinct genetic distributed in Costa Rica and western Panama isolated from lineages in central Panama, a pattern known as the ‘western Panama isthmus’ (WPI) break [[1]](https://paperpile.com/c/Z0W1m7/KOoQ9); Bagley et al. in review[[1]](https://paperpile.com/c/Z0W1m7/KOoQ9). The ‘frogs and fishes’ dataset contains 109 mitochondrial DNA sequences from 564 to 1877 bp in length, mostly of the cytochrome oxidase 1 gene, but also other genes, from 10–28 individuals from each of seven species/lineages split across the break, all obtained from GenBank. This sampling included at least 2 and up to 16 individuals sampled on either side of the WPI break. For comparative purposes, other than adding the β parameter and considering a broader range of priors, our analysis employed the same analytical approach and priors as those used by Bagley et al. (in review). For example, we used the same ABC model averaging approach [[2–4]](https://paperpile.com/c/Z0W1m7/3qIc4+pGWlV+O5GPe), and in our coalescent simulations, we also used HKY [[5]](https://paperpile.com/c/Z0W1m7/vCLej) models with gamma-distributed rate variation (Γ) and an estimated proportion of invariant sites (*I*) across taxa. However, our re-analysis considered a candidate set of 10 model classes (*M*_1_–*M*_10_), each consisting of one of two uniformly distributed priors on population divergence times (*τ*), the *τ* buffering parameter (β), ancestral population size (*θ*_A_), and daughter population size parameters (*θ*_D_; Table S1). This set of prior model classes allowed us to compare results of using low, moderate, and high β values ranging from 0.025 to 0.3 for the fishes, and from 0.05 to 0.6 for the frogs. As in Bagley et al. (in review), we simulated 5 million datasets from each prior model class, and then we graphically checked for efficient prior sampling using principal components analysis (PCA) on 1000 prior draws from each model class in R v3.2.3 [[6]](https://paperpile.com/c/Z0W1m7/nTM1b). Resulting estimates of Ω and *Ψ* were weighted by Bayesian model averaging [[3]](https://paperpile.com/c/Z0W1m7/pGWlV).

We conducted hypothesis testing by comparing posterior probabilities of the hyper-parameter estimates under a scenario of asynchronous diversification (Ω > 0.01; *Ψ* > 1) versus the alternative of simultaneous diversification (Ω ≤ 0.01; *Ψ=* 1) [[7–9]](https://paperpile.com/c/Z0W1m7/JEyLQ+xzefV+dtQxY) and subsequently calculating *B*_10_ Bayes factors under the parameter thresholds above while accounting for prior support for the hypotheses, using *B*_10_ “weight of evidence” criteria in Jeffreys [[10]](https://paperpile.com/c/Z0W1m7/lILFy) and Kass and Raftery [[11]](https://paperpile.com/c/Z0W1m7/pt04n). We estimated mean assemblage-wide divergence times by converting model-averaged *E*[*τ*] estimates (in coalescent units of 4*N*_ave_ generations) to absolute time (*T*_div_) using the equation *T*_div_ = *E*[*τ*] × (*θ*_ave_ / *μ*), where *μ* is the assumed mutation rate per site per generation and *θ*_ave_ (per site) is the mean of the upper *θ* prior. Conversions used mutation rates equivalent to 0.7% lineage^−1^ Myr^−1^ and 0.785% lineage^−1^ Myr^-1^, the median rates of uniform ‘frog rate’ and ‘fish rate’ priors used in recent BEAST analyses of the data in Bagley et al.’ (in review).

**Application to empirical data: Neotropical Butterflies**

Garzón-Orduña et al. [[12]](https://paperpile.com/c/Z0W1m7/1DBZQ) recently undertook a substantial analysis of Neotropical butterfly divergence. They reported divergence times of 131 taxon-pairs using an assumed mutation rate and pairwise distances of COI sequence data without accounting for gene tree / species tree discordance. They found that the majority of speciation events occurred during the Pleistocene and therefore suggest that the Pleistocene Refugium Hypothesis may be useful for explaining some or much of Neotropical butterfly diversity. To re-analyze these data, we obtained the sequence files from the corresponding author (personal communication). Besides the 131 taxon-pairs the original study also tested divergence between species triplets (pairs of sister species and their closest relative). We pruned out these species triplets, as well as many other species that did not have a well resolved closest relative, or for which the sequence data was not provided, resulting in a final dataset composed of 116 taxon-pairs (mean *n* per pair = 3.4; min. *n* per pair = 2; max. *n* per pair = 36). We visually inspected all alignments. When possible, alignment error was corrected manually, and when not possible we performed global realignment with MUSCLE [[13]](https://paperpile.com/c/Z0W1m7/agLGj). After verification, aligned fasta files were converted to IM with a custom python script (to be uploaded to github, along with the iPython notebook for reproducing all the computation), and then pre-processed for MTML-MsBayes using `convertIM.pl`. Keightley et al. have estimated *Heliconius* constant mutation rate of 1.9 × 10^-9^ per site per generation [[14]](https://paperpile.com/c/Z0W1m7/jvciU), but in order to maintain as much concordance as possible between our re-analysis and the original analysis we chose an upper bound for τ using the oldest observed pairwise gene tree divergence assuming 1.1% sequence divergence per lineage per million years [[15]](https://paperpile.com/c/Z0W1m7/KmmzC), following [[12]](https://paperpile.com/c/Z0W1m7/1DBZQ).

The vast majority of taxon-pairs in the dataset were composed of a single sample per taxon, limiting our analysis of the full data to using only the number of pairwise differences between species (*π_b_*). In order to have a different lens of the history of these taxa and to take advantage of the full information in the summary statistic vector we also analyzed a subset of the data consisting of only taxon-pairs with 5 or more samples per taxon (*n* = 3). Guided by our simulation investigation, for both the full and the subsampled datasets we used a uniform prior with order-independent sorting of the summary statistic vector and report results based on β = 0.0, 0.05, 0.01, and 0.1. Reference tables were generated by simulating 3 million samples from the hyper-prior and hyper-posterior distribution were sampled from 10000 samples retained via simple rejection. For the continuous hyperparameter summary Ω*,* we performed local linear regression followed by boundary transformation. We graphically checked goodness of fit of the models to the observed data by computing principal component and plotting the first principal components. We further tested goodness of fit by calculating the *D*_prior_ statistic as implemented in the *gfit()* function [[16]](https://paperpile.com/c/Z0W1m7/AJ9nD) of the 'abc' package [[17]](https://paperpile.com/c/Z0W1m7/FIZQi) for R [[6]](https://paperpile.com/c/Z0W1m7/nTM1b). After confirming goodness of fit, we analyze the results as above, calculating Bayes factors and estimating mean assemblage-wide divergence times.

For the reduced dataset with three taxon-pairs of butterflies, the posterior probability of Ψ provided strong support for asynchronous divergence between all three taxon pairs regardless of the value of β selected (Pr(Ψ = 3 | β = 0) = 0.69); Pr(Ψ = 3 | β = 0.01) = 0.59); Pr(Ψ = 3 | β = 0.05) = 0.68); Pr(Ψ = 3 | β = 0.1) = 0.63). This is consistent with our simulation experiment results showing low impact of chosen β values given only three taxon pairs. In all cases, the posterior probability of one shared divergence event (Ψ = 1) was nearly 0 for this dataset. The resulting Bayes factors comparing models of fully asynchronous divergence (Ψ = 3) to models including some synchronous divergence (Ψ < 3) show strong support for full asynchrony across all buffering regimes (BF(Ψ = 3, Ψ < 3) > 25 for all β) [[11]](https://paperpile.com/c/Z0W1m7/pt04n). Similarly, estimates of Ω strongly supported a model of idiosyncratic divergence with mode and 95% HPD not significantly differing across buffering regimes (Mode estimate of Ω: β(0) = 0.33; β(0.01) = 0.35; β(0.05) = 0.35; β (0.1) = 0.33). The lower bound of 95% HPD on the estimate of Ω for all values of β was > 0.18, indicating inference of considerable variability in divergence times.

The conspicuous feature of the reduced three taxon-pair butterfly analysis was the uniformity of results across widely different buffering regimes. Estimates of Ψ and Ω with β = 0 do not differ significantly from estimates with β = 0.1, and given β = 0.1, the Bayes factor for Ψ = 3 versus Ψ < 3, as well as the mode and 95% HPD of Ω indicate asynchronous divergence, suggesting that the taxon pairs diverged at considerably different times. This finding is in agreement with the divergence time estimates of Garzón‐Orduña et al. [[12]](https://paperpile.com/c/Z0W1m7/1DBZQ), who calculated uncorrected *p*-distances and estimated divergence times for *Ithioma* (*p-*distance: 0.004; divergence time: 0.17 My), *Hyposcada* (*p-*distance: 0.034; divergence time: 1.56 My) and *Parides* (*p-*distance: 0.076; divergence time: 3.46 My), the three taxon pairs used in our reduced dataset.

**Size of reference table**

We were also interested in investigating the impact of the size of reference tables on the bias and accuracy of sorted versus unsorted summary statistics vectors. Sorting significantly reduces the size of summary statistic space, allowing for reasonably accurate inference based on computationally tractable reference table sizes. On the other hand, inference based on unsorted summary statistics is predicted to require far greater sampling from the prior distribution to obtain similar power as the number of taxon-pairs increases. To test this, we simulated both 3 × 10^6^ and 5 × 10^7^ samples from the prior and repeated the PODS analysis for each of the three data configurations above. As above, after post-acceptance adjustment, we retained an approximate posterior of 1000 samples for both reference tables.

Sorting the summary statistics extracts information about the variability in divergence times while reducing the size of combinatorial sample space across simulations [[2,3]](https://paperpile.com/c/Z0W1m7/3qIc4+pGWlV). In practice, this allows us to reduce the size of the reference table while retaining reasonable accuracy in the estimation of Ω. While the condition of the exchangeability of summary statistics is not expected to be met with most empirical datasets, use of unsorted summary statistics are expected to require significantly larger reference tables to obtain equal power. The difference in the number of possible discrete models with sorted versus unsorted summary statistics under the 18 taxon-pair data configuration is substantial (385 models versus 6.8 × 10^11^ models, respectively). Given the probable unwieldiness of a reference table 9 orders of magnitude larger than that of our initial experiment we compare two sizes of reference tables, (3 × 10^6^ and 5 × 10^7^ samples from the prior) reasoning that increasing the size by one order of magnitude should provide measurable effects. For the each of the 3, 4, and 18 species-pair data configurations there is negligible improvement in estimation of both Ψ and Ω regardless of choices in sorting and/or hyperprior on Ψ (Table 1). RMSE is reduced by ~1% and there is no qualitative change in the performance with respect to sorting strategy or choice of prior on Ψ. While these finding are in agreement with [[18]](https://paperpile.com/c/Z0W1m7/wIadh) who detected no measurable improvement in performance with increasing prior sample sizes, we are cautious about a direct comparison as there are some key differences in sampling configurations and chosen widths of prior distributions. Given these results we find reference tables on the order of 3 × 10^6^ draws from the hyperprior represent a good tradeoff between accuracy and computational effort.

While sorting strongly outperforms unsorting for estimating Ω given reference table sizes that we consider computationally feasible, one might predict that unsorting would gradually obtain and then surpass the accuracy of sorting as the size of the reference table increases. Indeed, similar to the findings of [[19]](https://paperpile.com/c/Z0W1m7/l5PVy) we gained slight but detectable improvements in RMSE for both for Ψ and Ω by increasing the size of the reference tables by one order of magnitude (from 3 × 10^6^ to 5 × 10^7^). Although it is unclear whether and at what rate these improvements would continue to accumulate with continually increasing reference table sizes, expanding beyond the sizes we considered is probably a trade-off between increasing computational effort and marginal gains in accuracy of inference.

**Literature Cited**

[1. Bagley JC, Johnson JB. Phylogeography and biogeography of the lower Central American Neotropics: diversification between two continents and between two seas. Biol. Rev. Camb. Philos. Soc. 2014;89:767–90.](http://paperpile.com/b/Z0W1m7/KOoQ9)

[2. Huang W, Takebayashi N, Qi Y, Hickerson MJ. MTML-msBayes: approximate Bayesian comparative phylogeographic inference from multiple taxa and multiple loci with rate heterogeneity. BMC Bioinformatics. 2011;12:1.](http://paperpile.com/b/Z0W1m7/3qIc4)

[3. Hickerson MJ, Stone GN, Lohse K, Demos TC, Xie X, Landerer C, et al. Recommendations for using msBayes to incorporate uncertainty in selecting an abc model prior: a response to Oaks et al. Evolution. 2014;68:284–94.](http://paperpile.com/b/Z0W1m7/pGWlV)

[4. Bagley JC. Understanding the diversification of Central American freshwater fishes using comparative phylogeography and species delimitation. Brigham Young University; 2014. Available from:](http://paperpile.com/b/Z0W1m7/O5GPe) <http://scholarsarchive.byu.edu/etd/5296/>

[5. Hasegawa M, Kishino H, Yano T. Dating of the human-ape splitting by a molecular clock of mitochondrial DNA. J. Mol. Evol. 1985;22:160–74.](http://paperpile.com/b/Z0W1m7/vCLej)

[6. Team RC. The R project for statistical computing. Available at www.R-project.org](http://paperpile.com/b/Z0W1m7/nTM1b).

[7. Hickerson MJ, Stahl EA, Lessios HA. Test for simultaneous divergence using approximate Bayesian computation. Evolution. 2006;60:2435–53.](http://paperpile.com/b/Z0W1m7/JEyLQ)

[8. Hickerson MJ, Stahl E, Takebayashi N. msBayes: pipeline for testing comparative phylogeographic histories using hierarchical approximate Bayesian computation. BMC Bioinformatics. 2007;8:268.](http://paperpile.com/b/Z0W1m7/xzefV)

[9. Bell RC, MacKenzie JB, Hickerson MJ, Chavarría KL, Cunningham M, Williams S, et al. Comparative multi-locus phylogeography confirms multiple vicariance events in co-distributed rainforest frogs. Proc. Biol. Sci. 2012;279:991–9.](http://paperpile.com/b/Z0W1m7/dtQxY)

[10. Harold Jeffreys S. Theory of probability. Oxford: Clarendon Press; 1961;](http://paperpile.com/b/Z0W1m7/lILFy)

[11. Kass RE, Raftery A. Bayes factors. J. Am. Stat. Assoc. 1995;90:773–95.](http://paperpile.com/b/Z0W1m7/pt04n)

[12. Garzón‐Orduña IJ, Benetti‐Longhini JE, Brower AV. Timing the diversification of the Amazonian biota: butterfly divergences are consistent with Pleistocene refugia. J. Biogeog.](http://paperpile.com/b/Z0W1m7/1DBZQ) 2014. [doi:10.1111/jbi.12330/full](http://onlinelibrary.wiley.com/doi/10.1111/jbi.12330/full)

[13. Edgar RC. MUSCLE: multiple sequence alignment with high accuracy and high throughput. Nucleic Acids Res. 2004;32:1792–7.](http://paperpile.com/b/Z0W1m7/agLGj)

[14. Keightley PD, Pinharanda A, Ness RW, Simpson F, Dasmahapatra KK, Mallet J, et al. Estimation of the spontaneous mutation rate in Heliconius melpomene. Mol. Biol. Evol. 2015;32:239–43.](http://paperpile.com/b/Z0W1m7/jvciU)

[15. Brower AV. Rapid morphological radiation and convergence among races of the butterfly Heliconius erato inferred from patterns of mitochondrial DNA evolution. Proc. Natl. Acad. Sci. 1994;91:6491–5.](http://paperpile.com/b/Z0W1m7/KmmzC)

[16. Lemaire L, Jay F, Lee I-H, Csilléry K, Blum MGB. Goodness-of-fit statistics for approximate Bayesian computation. arXiv preprint. 2016.](http://paperpile.com/b/Z0W1m7/AJ9nD) [arxiv.org/abs/1601.04096](http://arxiv.org/abs/1601.04096)

[17. Csilléry K, François O, Blum MGB. abc: an R package for approximate Bayesian computation (ABC). Methods Ecol. Evol. 2012;3:475–9.](http://paperpile.com/b/Z0W1m7/FIZQi)

[18. Oaks JR, Sukumaran J, Esselstyn JA, Linkem CW, Siler CD, Holder MT, et al. Evidence for climate-driven diversification? A caution for interpreting ABC inferences of simultaneous historical events. Evolution. 2013;67:991–1010.](http://paperpile.com/b/Z0W1m7/wIadh)

[19. Papadopoulou A, Knowles LL. Species-specific responses to island connectivity cycles: refined models for testing phylogeographic concordance across a Mediterranean Pleistocene Aggregate Island Complex. Mol. Ecol. 2015;24:4252–68.](http://paperpile.com/b/Z0W1m7/l5PVy)

**Supporting Tables and Figures**

**Table S1**. Prior model classes and β values for reanalysis of Panamanian Fish and Frog data.

|  | P(*τ*) | P(θ_D_) | P(θ_A_) | β |  |  |
| --- | --- | --- | --- | --- | --- | --- |
|  |  |  |  |  |  |  |
| *M_1_* | ~U(0,1.75)_Frogs_ ~U(0,0.8)_Fish_ | ~U(0,0.1) | ~U(0,0.25) | 0.05 |  |  |
| *M_2_* | ~U(0,1.75)_Frogs_ ~U(0,0.8)_Fish_ | ~U(0,0.1) | ~U(0,0.25) | 0.1 |  |  |
| *M_3_* | ~U(0,1.75)_Frogs_ ~U(0,0.8)_Fish_ | ~U(0,0.1) | ~U(0,0.25) | 0.2 |  |  |
| *M_4_* | ~U(0,1.75)_Frogs_ ~U(0,0.8)_Fish_ | ~U(0,0.1) | ~U(0,0.25) | 0.3 |  |  |
| *M_5_* | ~U(0,1.75)_Frogs_ ~U(0,0.8)_Fish_ | ~U(0,0.1) | ~U(0,0.25) | 0.6 |  |  |
| *M_6_* | ~U(0,1.75)_Frogs_ ~U(0,0.8)_Fish_ | ~U(0,0.1) | ~U(0,0.5) | 0.05 |  |  |
| *M_7_* | ~U(0,1.75)_Frogs_ ~U(0,0.8)_Fish_ | ~U(0,0.1) | ~U(0,0.5) | 0.1 |  |  |
| *M_8_* | ~U(0,1.75)_Frogs_ ~U(0,0.8)_Fish_ | ~U(0,0.1) | ~U(0,0.5) | 0.2 |  |  |
| *M_9_* | ~U(0,1.75)_Frogs_ ~U(0,0.8)_Fish_ | ~U(0,0.1) | ~U(0,0.5) | 0.3 |  |  |
| *M_10_* | ~U(0,1.75)_Frogs_ ~U(0,0.8)_Fish_ | ~U(0,0.1) | ~U(0,0.5) | 0.6 |  |  |

Parameters for two prior model classes for each of five values of β used to reanalyze two separate datasets testing simultaneous divergence across the Western Panama isthmus (WPI). The subscripts on values of P(*τ*) indicate the prior range on divergence times for the Panamanian fish (3 taxon-pairs) and frog (4 taxon-pairs) data configurations for each of the ten models (*M_1_*–*M_10_*). All other prior ranges and β values were shared across each of the ten models for both data configurations.

**Table S2**. RMSE on Ψ and Ω with mismatched buffering of priors and PODS.

| **β** | Buffered Prior  unbuffered PODS  Ψ | Buffered Prior  unbuffered PODS  Ω | Unbuffered Prior  buffered PODS  Ψ | Unbuffered Prior  buffered PODS  Ω |
| --- | --- | --- | --- | --- |
| 0 | 4.443 | 0.0314 | 4.443 | 0.0314 |
| 0.01 | 3.864 | 0.0349 | 4.52 | 0.0331 |
| 0.05 | 5.647 | 0.0281 | 6.413 | 0.0314 |
| 0.1 | 5.863 | 0.0381 | 7.986 | 0.034 |

Mean RMSE in estimation of Ψ and Ω averaged across 100 PODS for the 18 taxon-pair data configuration. These experiments were conducted with a uniform prior on Ψ, sorted summary statistics vectors, and 3 × 10^6^ samples from the prior distribution. Results are reported for inference of key parameters with a mismatch in β applied to the prior and the PODS. Buffered Prior

unbuffered PODS: Varying β values were applied to the prior and the PODS were sampled freely. Unbuffered Prior buffered PODS: Varying β values were applied to the PODS and the priors were sampled freely.

**Figure S1**


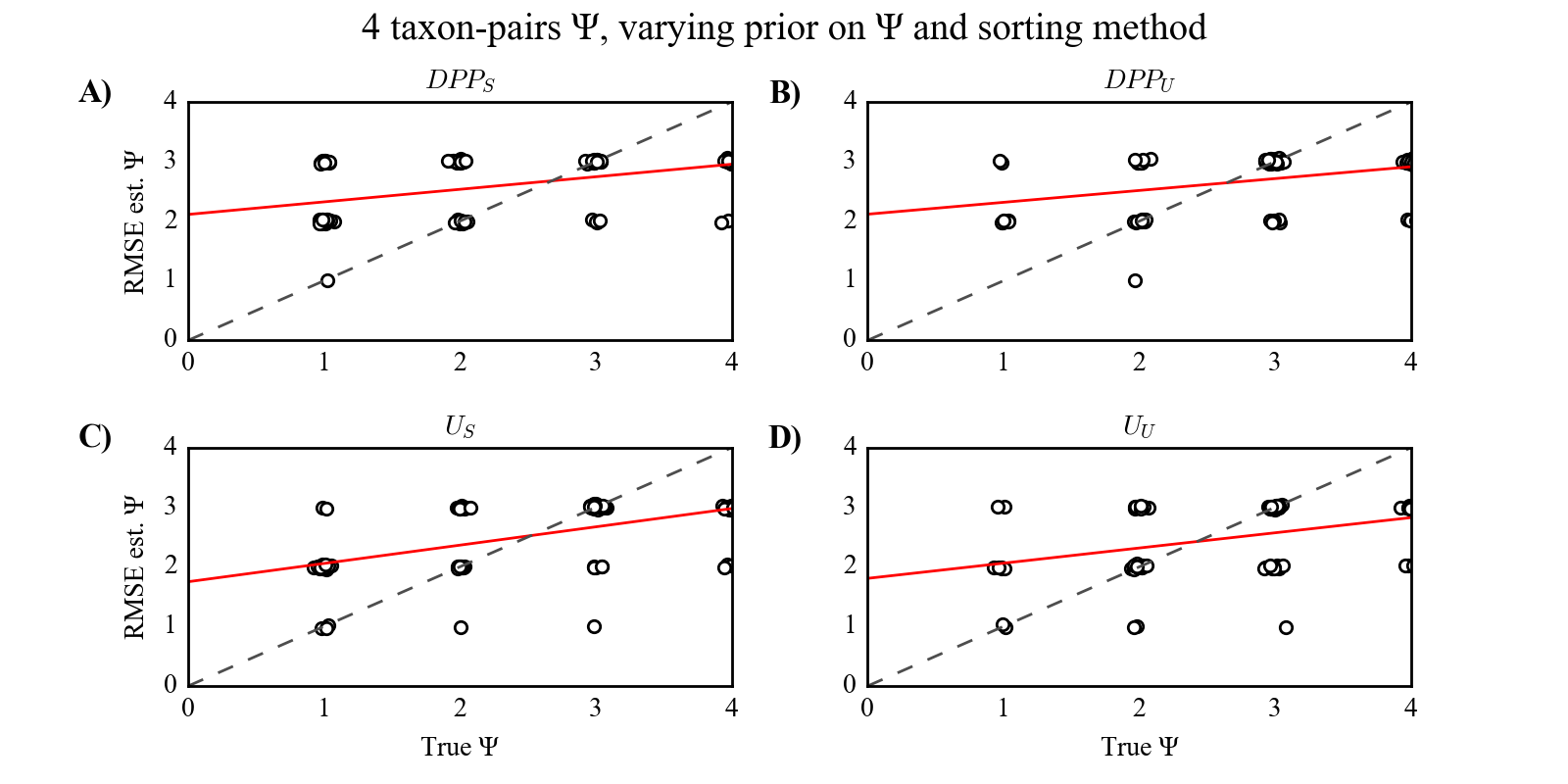


Scatterplots of true versus estimated values of Ψ for 100 PODS under different models of prior distribution on Ψ and applying different sorting strategies for the 4 taxon-pair data configuration. PODS were analyzed using reference tables composed of 3 × 10^6^ samples from the prior. Points in the plot are slightly perturbed to visualize the number of points for each estimate. The dashed line is the identity line, and the red line is a simple linear regression of estimated Ψ onto true Ψ. A) Dirichlet-process prior with sorted summary statistics. B) Dirichlet-process prior with unsorted summary statistics. C) Uniform prior with sorted summary statistics. D) Uniform prior with unsorted summary statistics.

**Figure S2**


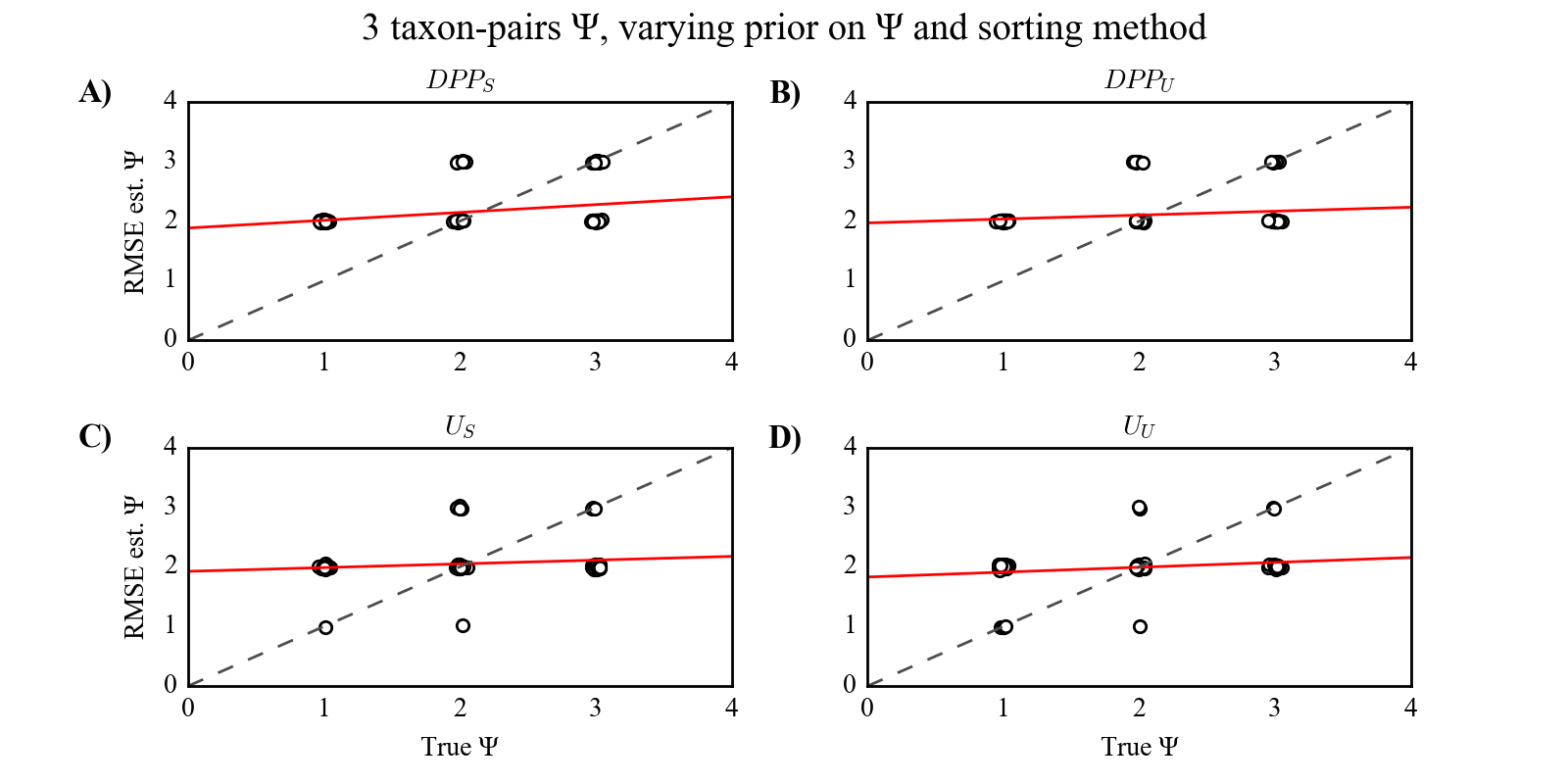


Scatterplots of true versus estimated values of Ψ for 100 PODS under different models of prior distribution on Ψ and applying different sorting strategies for the 3 taxon-pair data configuration. PODS were analyzed using reference tables composed of 3 × 10^6^ samples from the prior. Points in the plot are slightly perturbed to visualize the number of points for each estimate. The dashed line is the identity line, and the red line is a simple linear regression of estimated Ψ onto true Ψ. A) Dirichlet-process prior with sorted summary statistics. B) Dirichlet-process prior with unsorted summary statistics. C) Uniform prior with sorted summary statistics. D) Uniform prior with unsorted summary statistics.

Figure S3


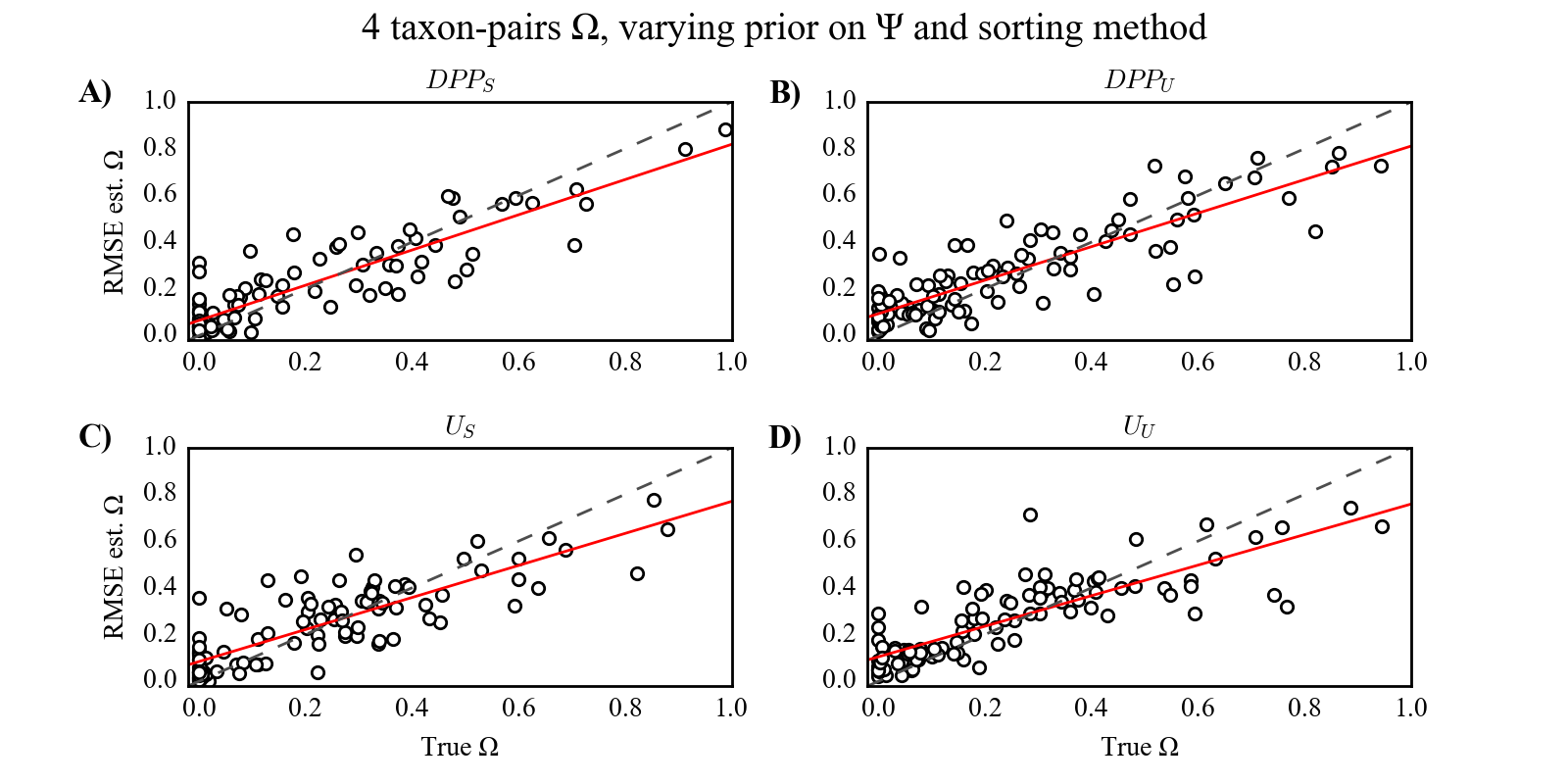


Scatterplots of true versus estimated values of Ω for 100 PODS under different models of prior distribution on Ψ and applying different sorting strategies for the 4 taxon-pair data configuration. PODS were analyzed using reference tables composed of 3 × 10^6^ samples from the prior. The dashed line is the identity line, and the red line is a simple linear regression of estimated Ω onto true Ω. A) Dirichlet-process prior with sorted summary statistics. B) Dirichlet-process prior with unsorted summary statistics. C) Uniform prior with sorted summary statistics. D) Uniform prior with unsorted summary statistics.

Figure S4


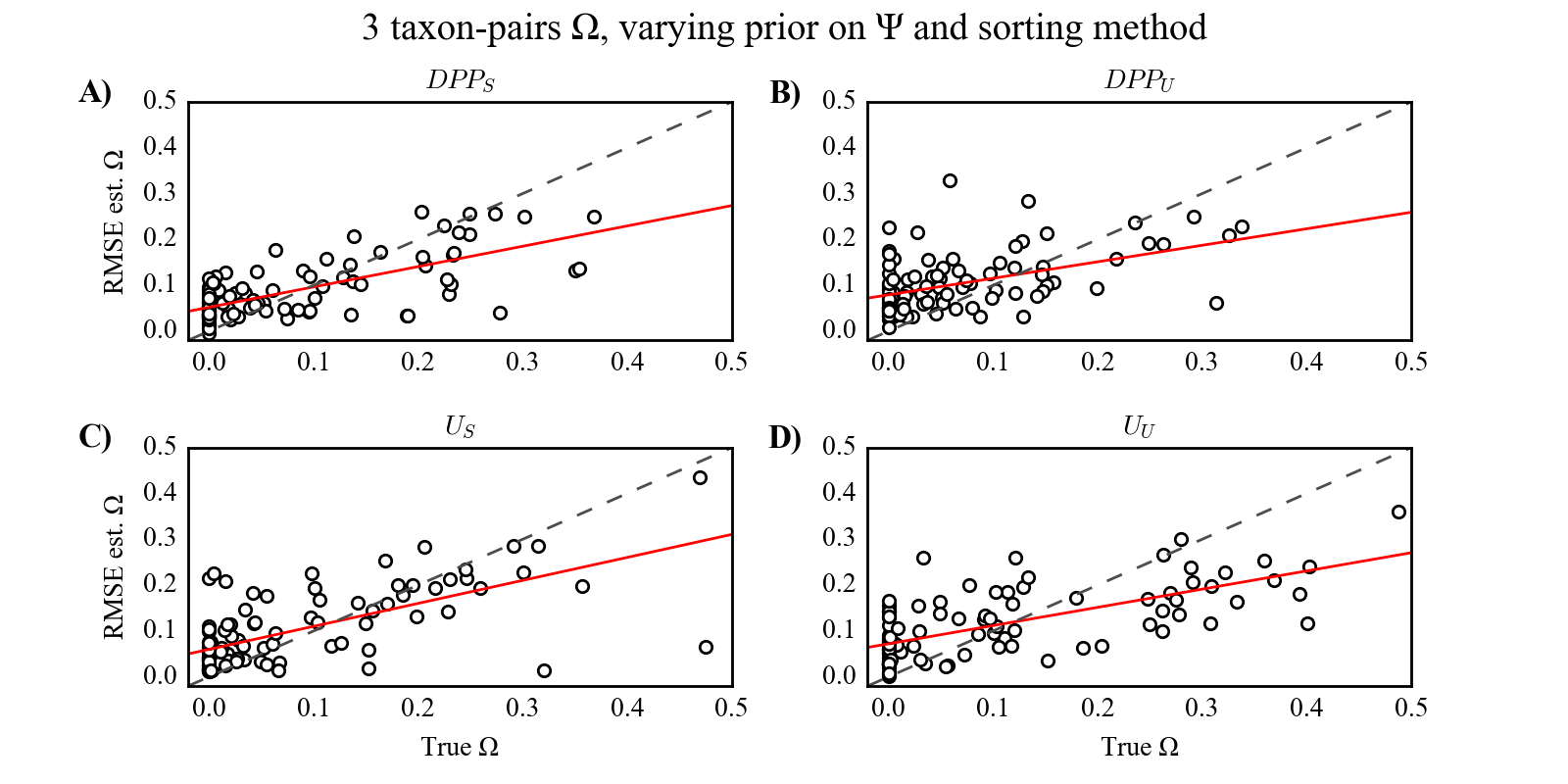


Scatterplots of true versus estimated values of Ω for 100 PODS under different models of prior distribution on Ψ and applying different sorting strategies for the 3 taxon-pair data configuration. PODS were analyzed using reference tables composed of 3 × 10^6^ samples from the prior. The dashed line is the identity line, and the red line is a simple linear regression of estimated Ω onto true Ω. A) Dirichlet-process prior with sorted summary statistics. B) Dirichlet-process prior with unsorted summary statistics. C) Uniform prior with sorted summary statistics. D) Uniform prior with unsorted summary statistics.

Figure S5


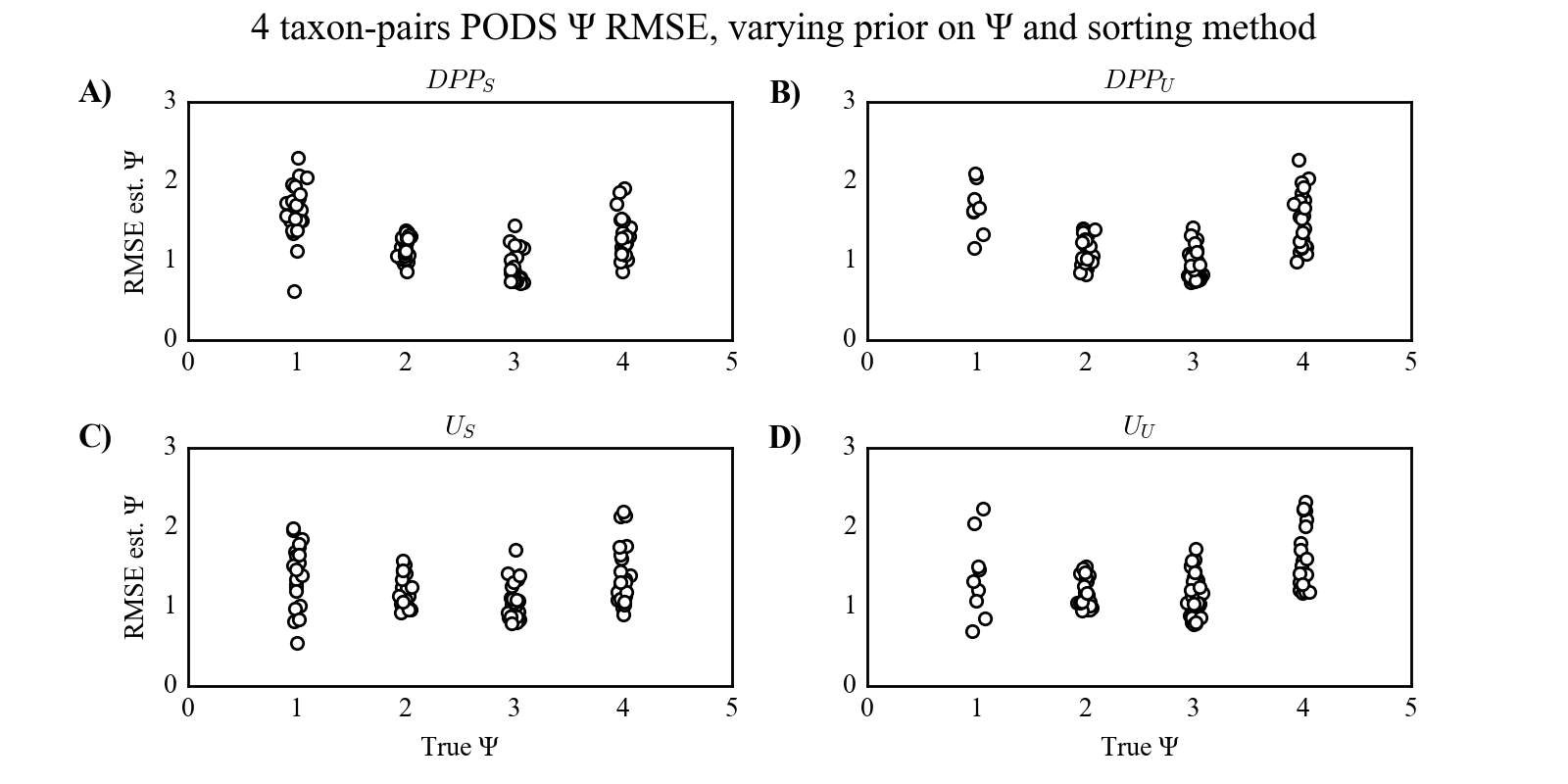


Scatterplots of RMSE in estimation of Ψ as a function of true Ψ for 100 PODS under different models of prior distribution on Ψ and applying different sorting strategies for the 4 taxon-pair data configuration. PODS were analyzed using reference tables composed of 3 × 10^6^ samples from the prior. Points in the plot are slightly perturbed to visualize the number of points for each estimate. A) Dirichlet-process prior with sorted summary statistics. B) Dirichlet-process prior with unsorted summary statistics. C) Uniform prior with sorted summary statistics. D) Uniform prior with unsorted summary statistics.

Figure S6


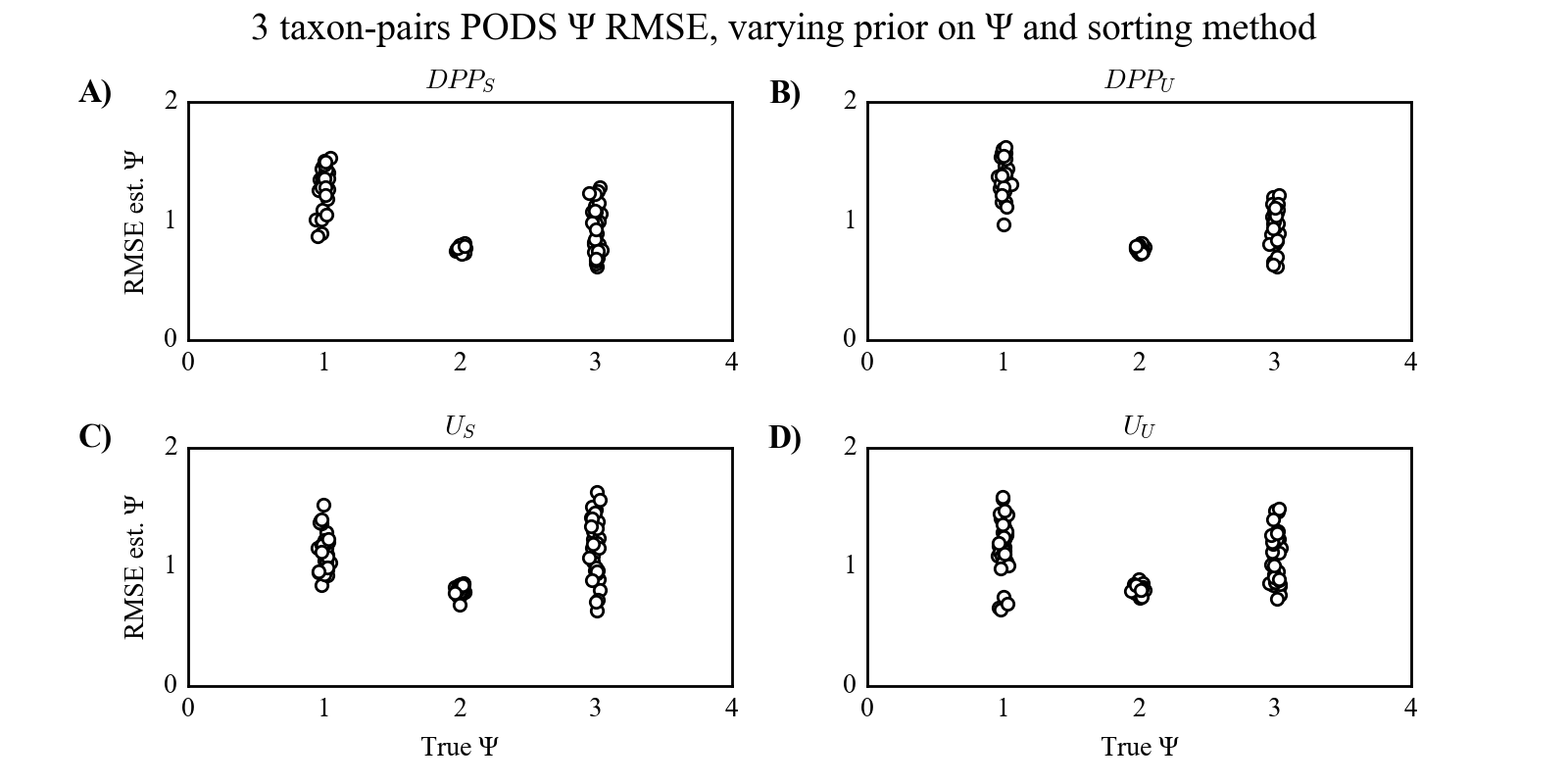


Scatterplots of RMSE in estimation of Ψ as a function of true Ψ for 100 PODS under different models of prior distribution on Ψ and applying different sorting strategies for the 3 taxon-pair data configuration. PODS were analyzed using reference tables composed of 3 × 10^6^ samples from the prior. Points in the plot are slightly perturbed to visualize the number of points for each estimate. A) Dirichlet-process prior with sorted summary statistics. B) Dirichlet-process prior with unsorted summary statistics. C) Uniform prior with sorted summary statistics. D) Uniform prior with unsorted summary statistics.

Figure S7


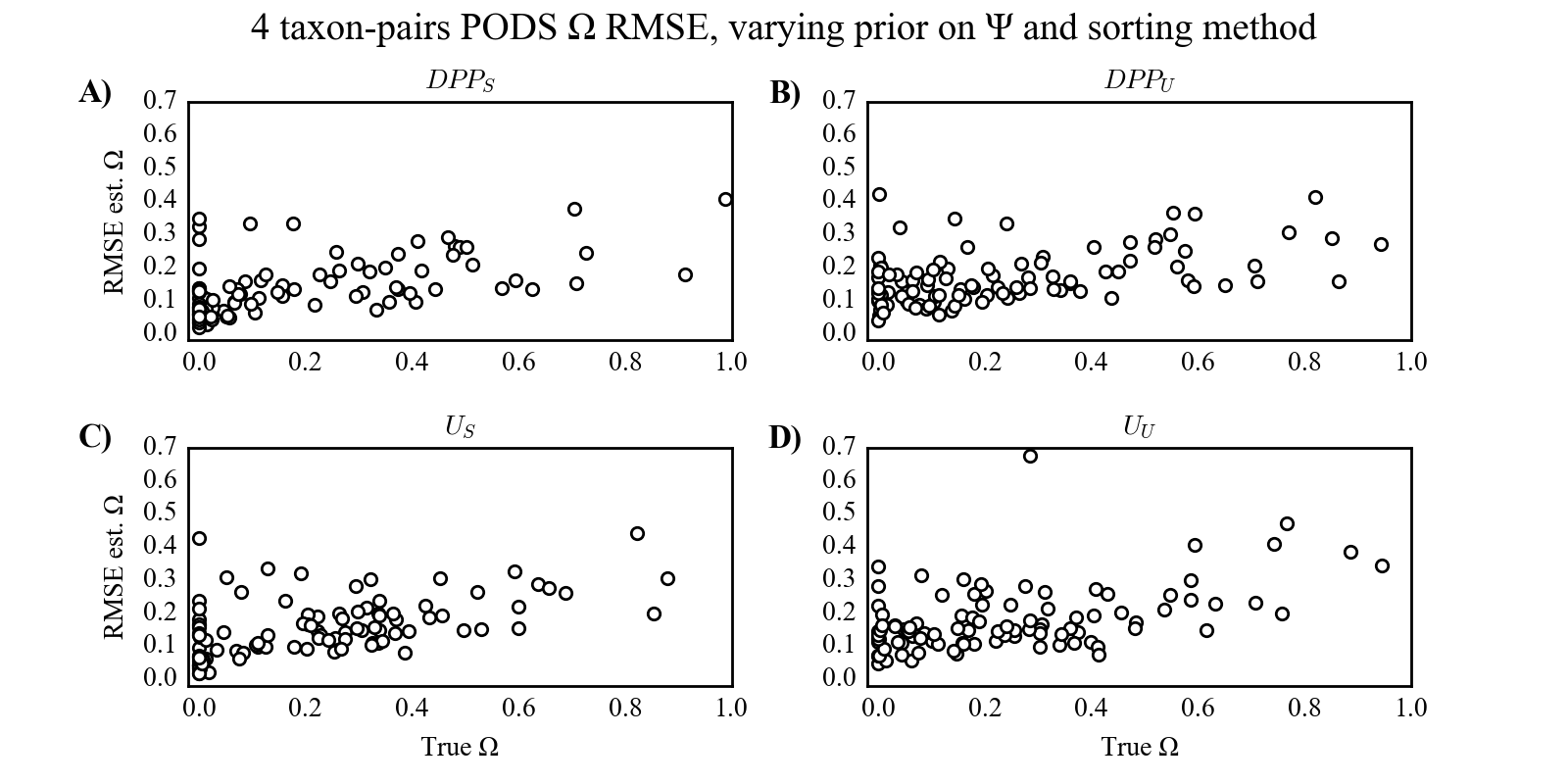


Scatterplots of RMSE in estimation of Ω as a function of true Ω for 100 PODS under different models of prior distribution on Ψ and applying different sorting strategies for the 4 taxon-pair data configuration. PODS were analyzed using reference tables composed of 3 × 10^6^ samples from the prior. A) Dirichlet-process prior with sorted summary statistics. B) Dirichlet-process prior with unsorted summary statistics. C) Uniform prior with sorted summary statistics. D) Uniform prior with unsorted summary statistics.

Figure S8


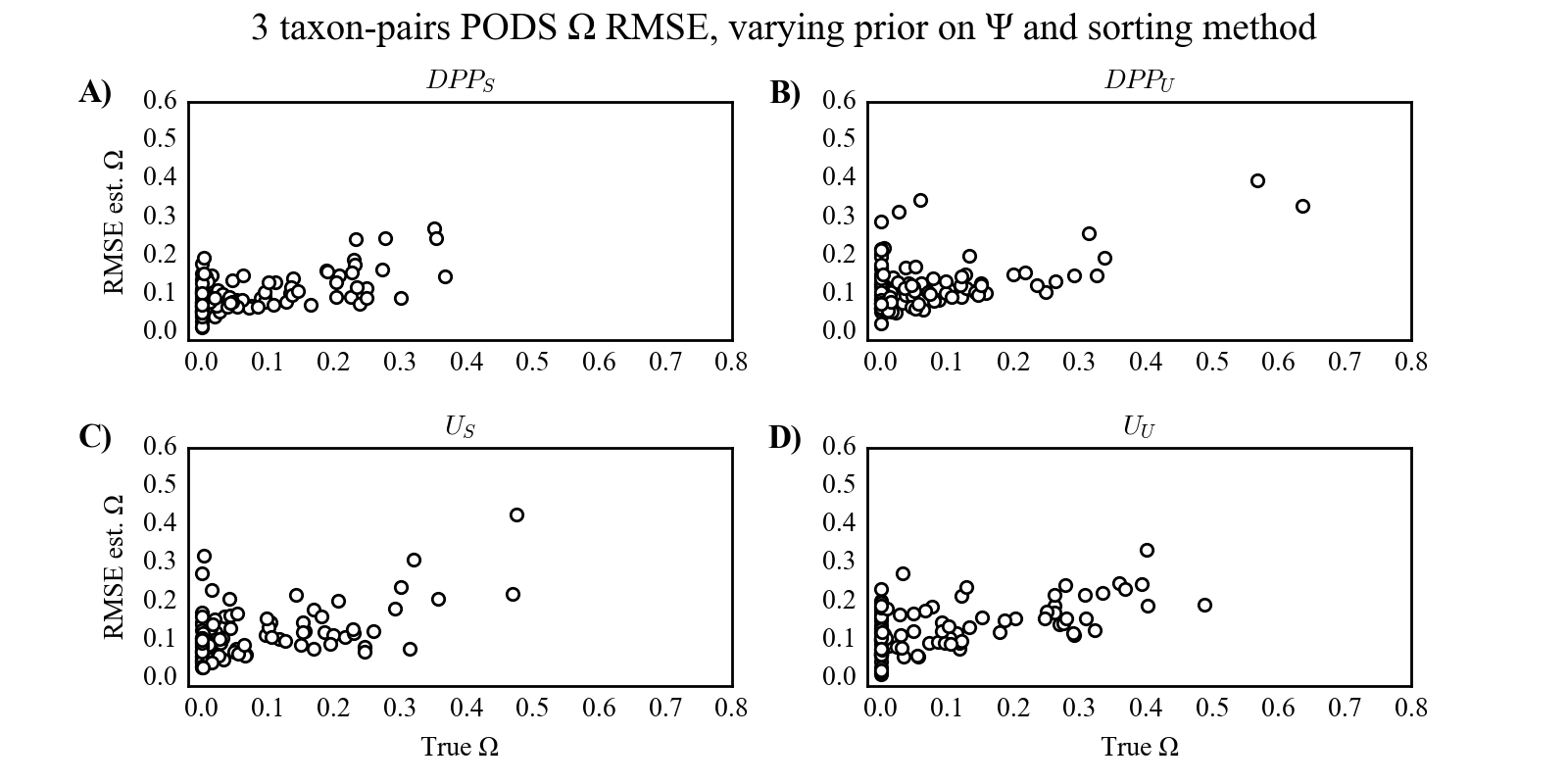


Scatterplots of RMSE in estimation of Ω as a function of true Ω for 100 PODS under different models of prior distribution on Ψ and applying different sorting strategies for the 3 taxon-pair data configuration. PODS were analyzed using reference tables composed of 3 × 10^6^ samples from the prior. A) Dirichlet-process prior with sorted summary statistics. B) Dirichlet-process prior with unsorted summary statistics. C) Uniform prior with sorted summary statistics. D) Uniform prior with unsorted summary statistics.

Figure S9


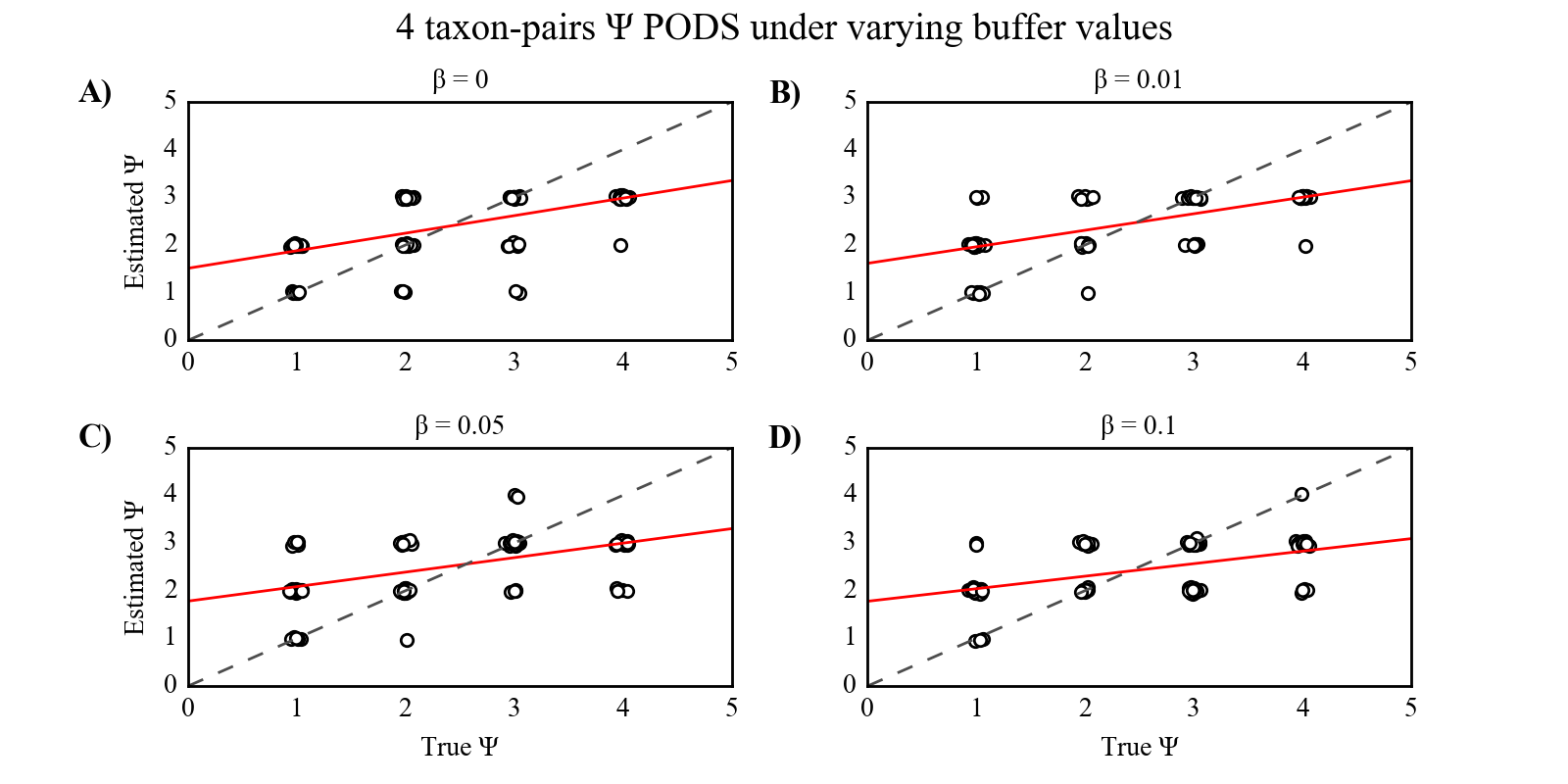


Scatterplots of true versus estimated values of Ψ for 100 PODS across different buffering regimes for the 4 taxon-pair data configuration. PODS were simulated and analyzed with a uniform prior on Ψ and sorted summary statistics using reference tables composed of 3 × 10^6^ samples from the prior. Points in the plot are slightly perturbed to visualize the number of points for each estimate. The dashed line is the identity line, and the red line is a simple linear regression of estimated Ψ onto true Ψ. A) β = 0 B) β = 0.01 C) β = 0.05 D) β = 0.1

Figure S10


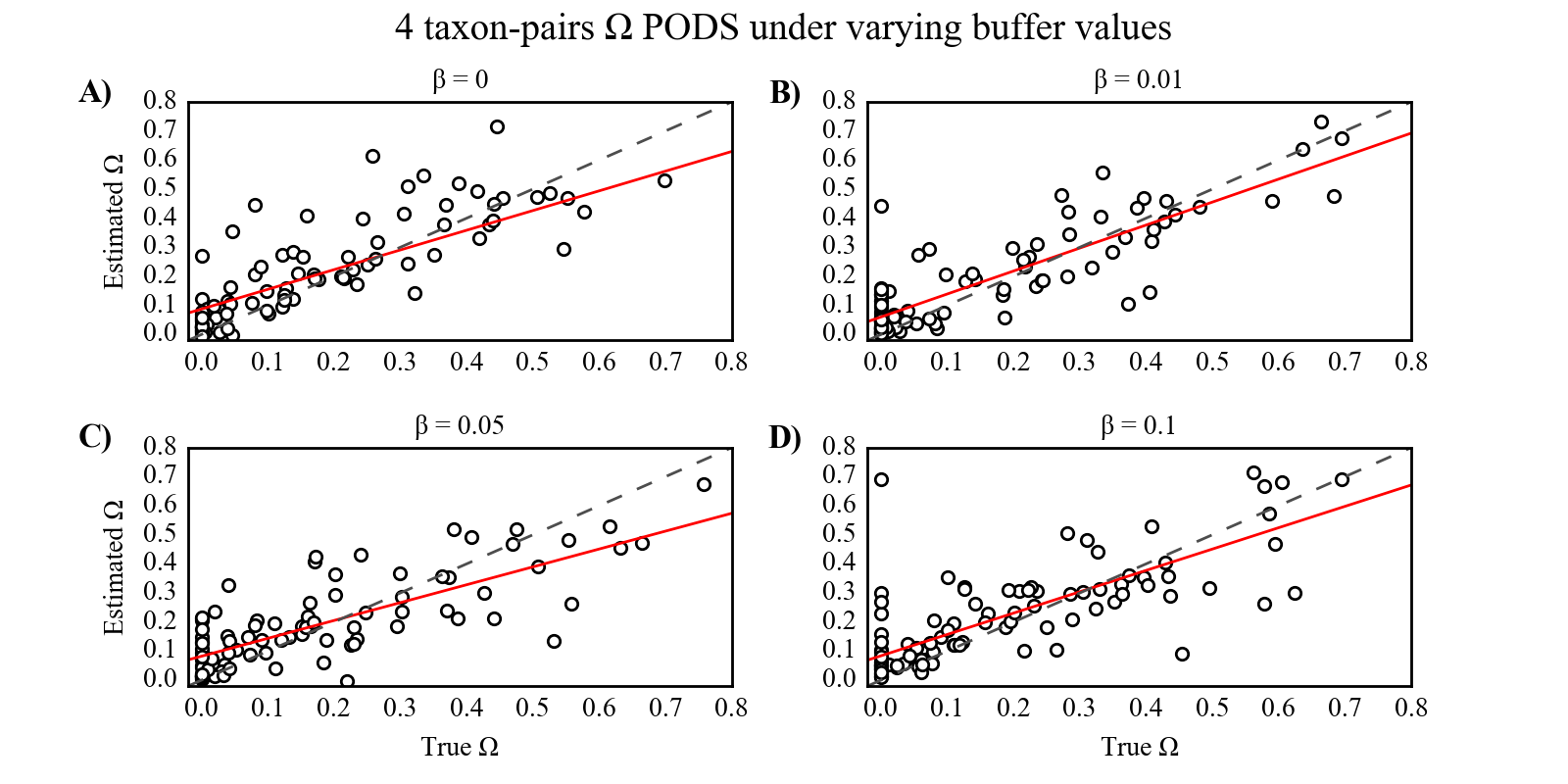


Scatterplots of true versus estimated values of Ω for 100 PODS across different buffering regimes for the 4 taxon-pair data configuration. PODS were simulated and analyzed with a uniform prior on Ψ and sorted summary statistics using reference tables composed of 3 × 10^6^ samples from the prior. The dashed line is the identity line, and the red line is a simple linear regression of estimated Ω onto true Ω. A) β = 0 B) β = 0.01 C) β = 0.05 D) β = 0.1

Figure S11


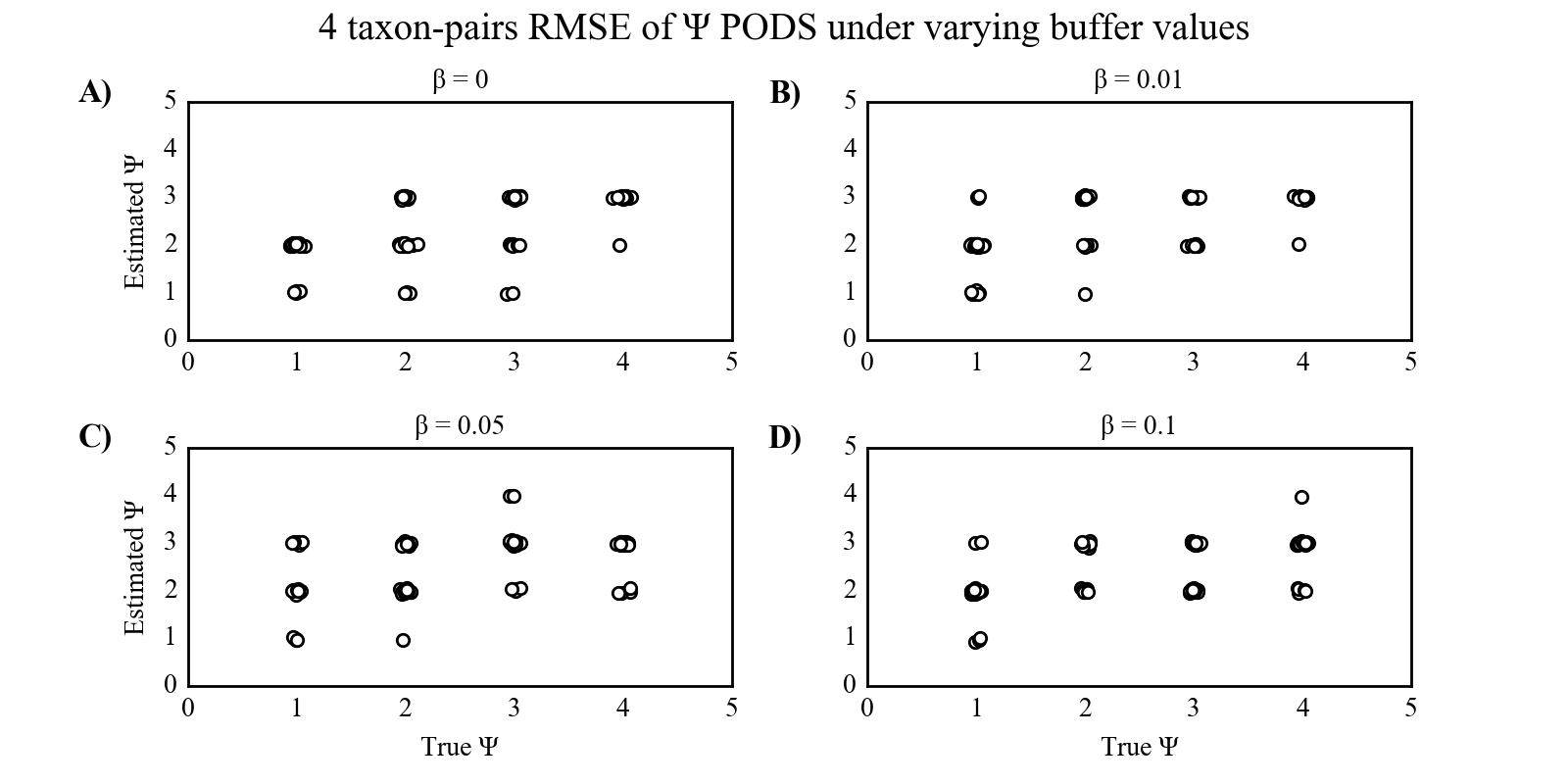


Scatterplots of RMSE in estimation of Ψ as a function of true Ψ for 100 PODS across different buffering regimes for the 4 taxon-pair data configuration. PODS were simulated and analyzed with a uniform prior on Ψ and sorted summary statistics using reference tables composed of 3 × 10^6^ samples from the prior. Points in the plot are slightly perturbed to visualize the number of points for each estimate. A) β = 0 B) β = 0.01 C) β = 0.05 D) β = 0.1

Figure S12


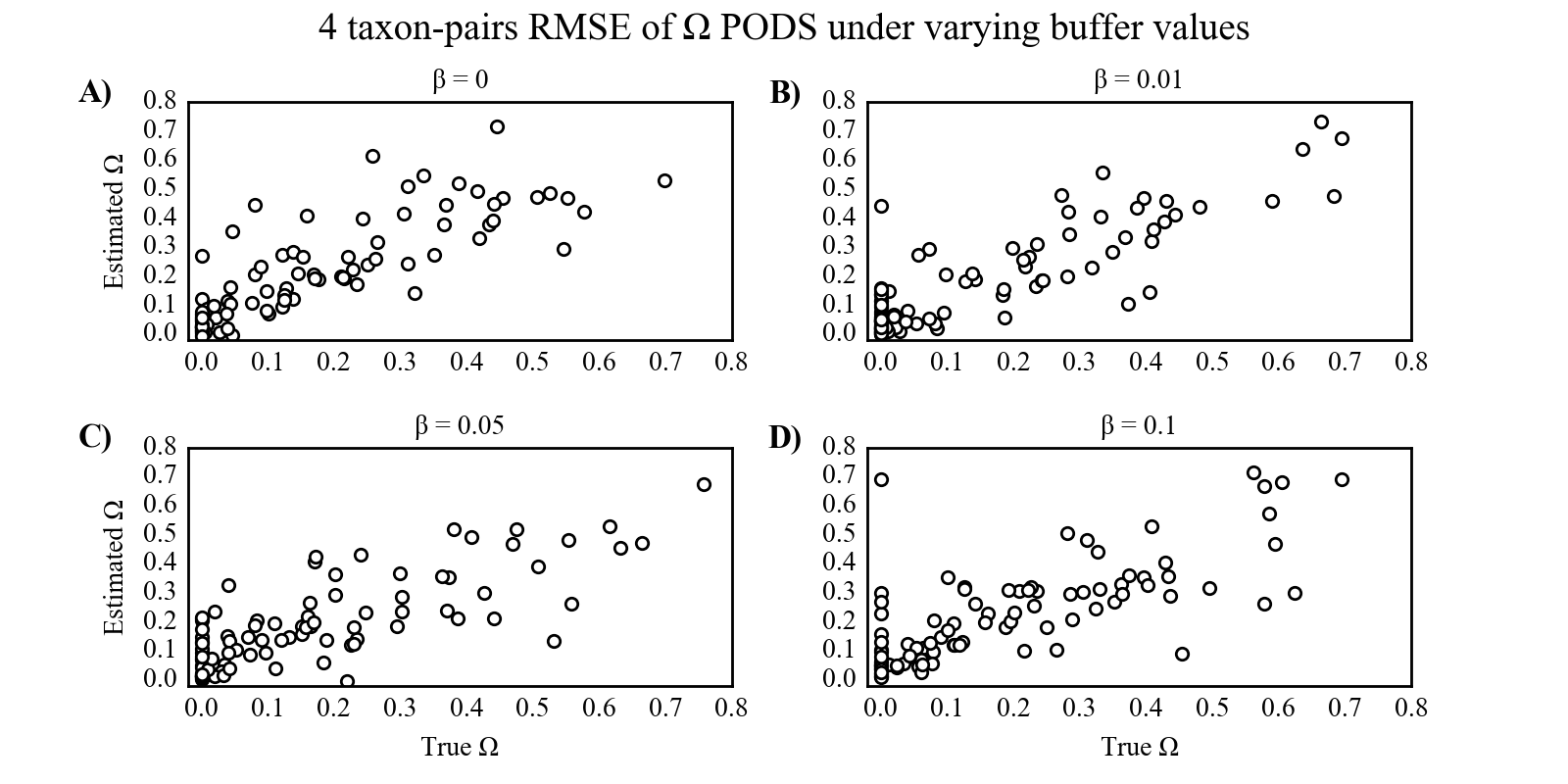


Scatterplots of RMSE in estimation of Ω as a function of true Ω for 100 PODS across different buffering regimes for the 4 taxon-pair data configuration. PODS were simulated and analyzed with a uniform prior on Ψ and sorted summary statistics using reference tables composed of 3 × 10^6^ samples from the prior. A) β = 0 B) β = 0.01 C) β = 0.05 D) β = 0.1

Figure S13


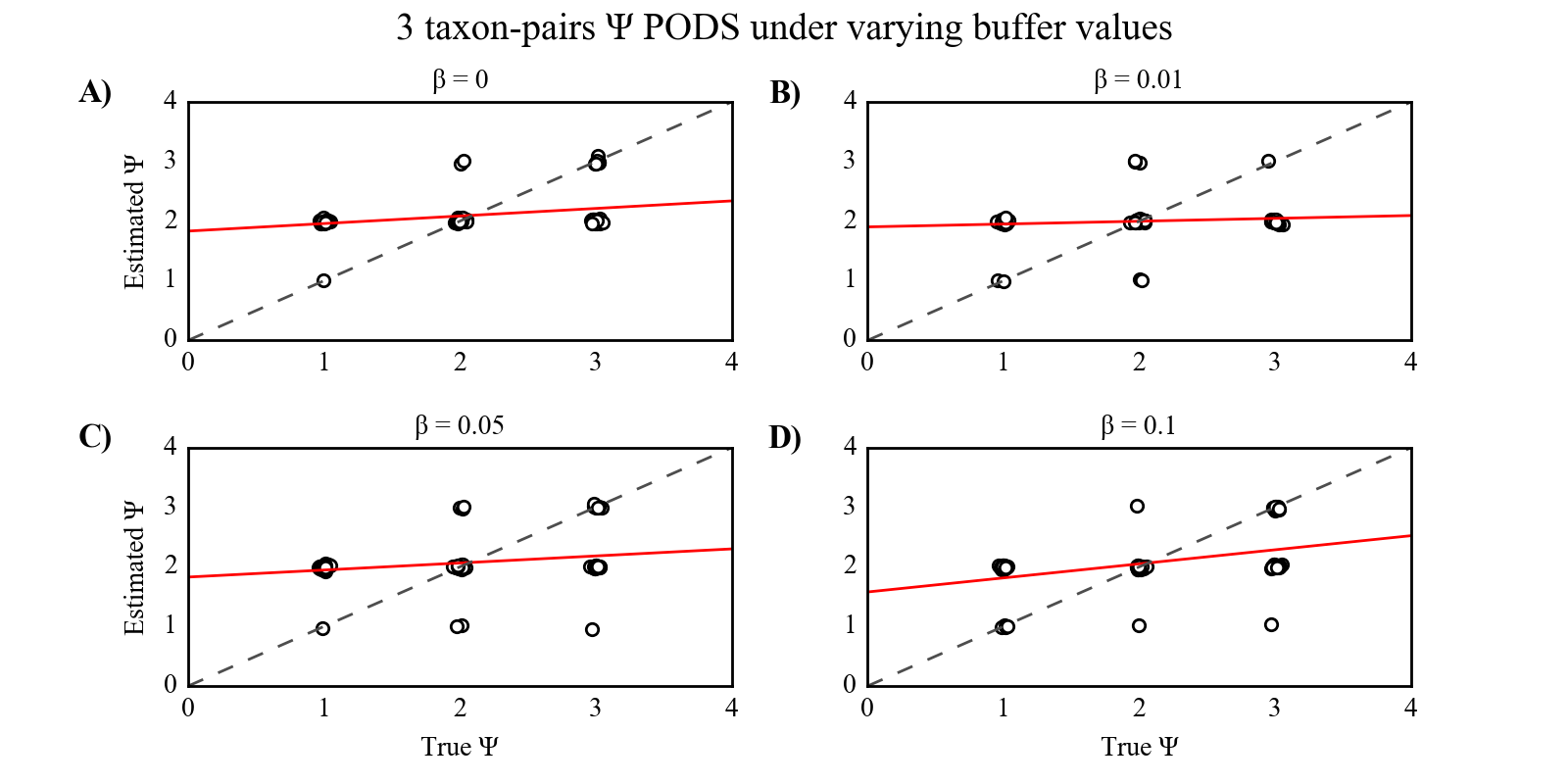


Scatterplots of true versus estimated values of Ψ for 100 PODS across different buffering regimes for the 3 taxon-pair data configuration. PODS were simulated and analyzed with a uniform prior on Ψ and sorted summary statistics using reference tables composed of 3 × 10^6^ samples from the prior. Points in the plot are slightly perturbed to visualize the number of points for each estimate. The dashed line is the identity line, and the red line is a simple linear regression of estimated Ψ onto true Ψ. A) β = 0 B) β = 0.01 C) β = 0.05 D) β = 0.1

Figure S14


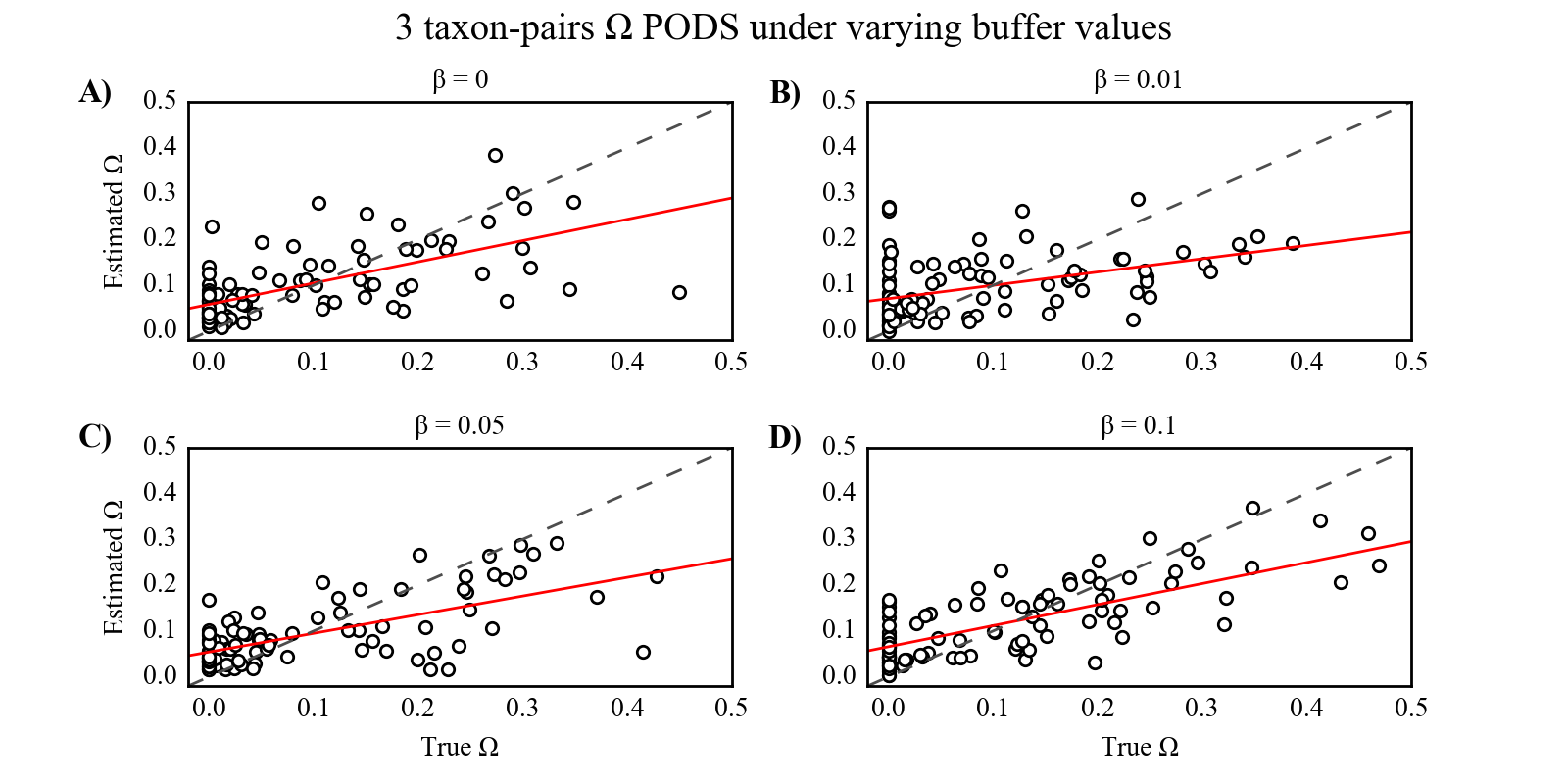


Scatterplots of true versus estimated values of Ω for 100 PODS across different buffering regimes for the 4 taxon-pair data configuration. PODS were simulated and analyzed with a uniform prior on Ψ and sorted summary statistics using reference tables composed of 3 × 10^6^ samples from the prior. The dashed line is the identity line, and the red line is a simple linear regression of estimated Ω onto true Ω. A) β = 0 B) β = 0.01 C) β = 0.05 D) β = 0.1

Figure S15


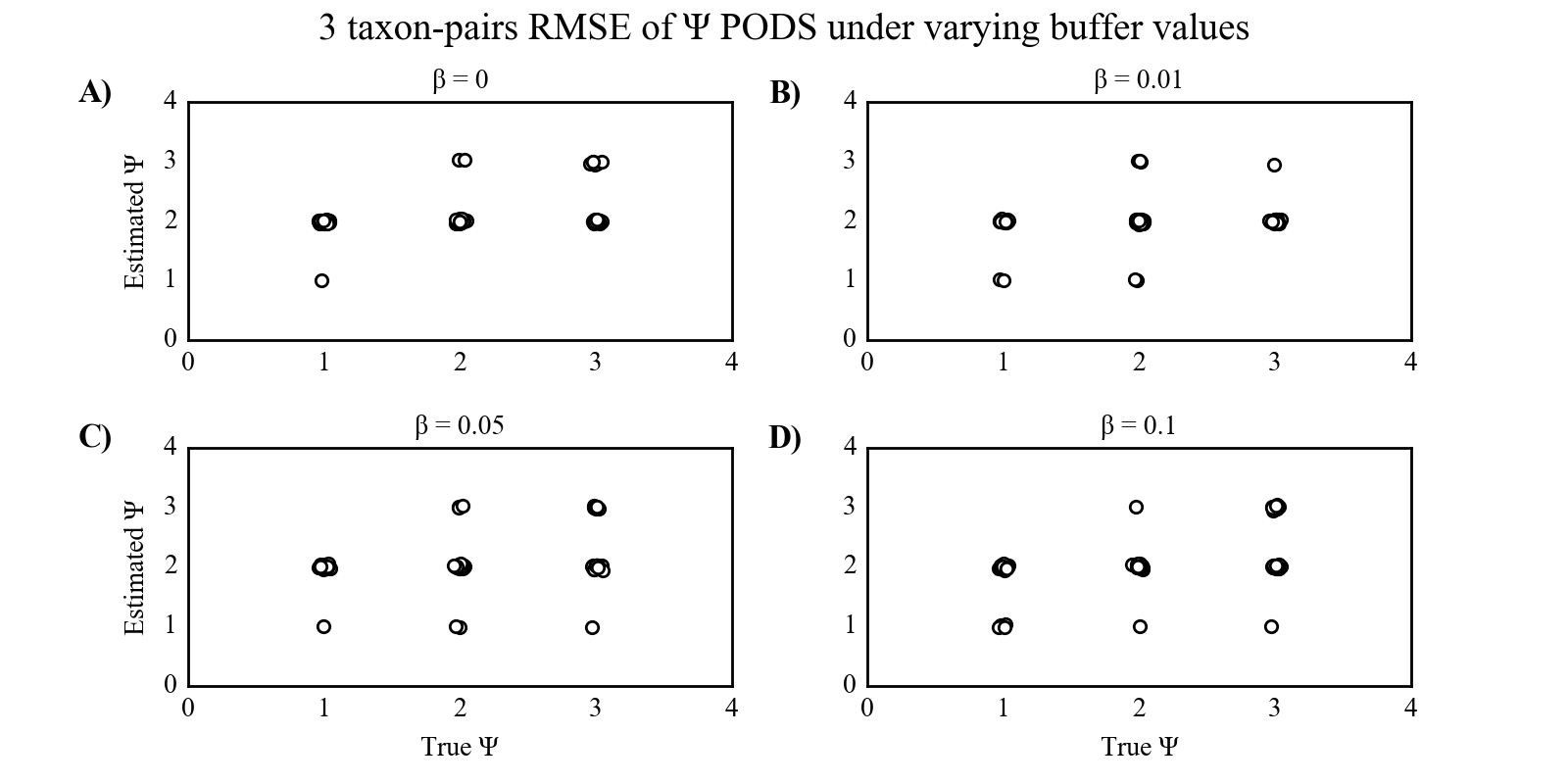


Scatterplots of RMSE in estimation of Ψ as a function of true Ψ for 100 PODS across different buffering regimes for the 3 taxon-pair data configuration. PODS were simulated and analyzed with a uniform prior on Ψ and sorted summary statistics using reference tables composed of 3 × 10^6^ samples from the prior. Points in the plot are slightly perturbed to visualize the number of points for each estimate. A) β = 0 B) β = 0.01 C) β = 0.05 D) β = 0.1

Figure S16


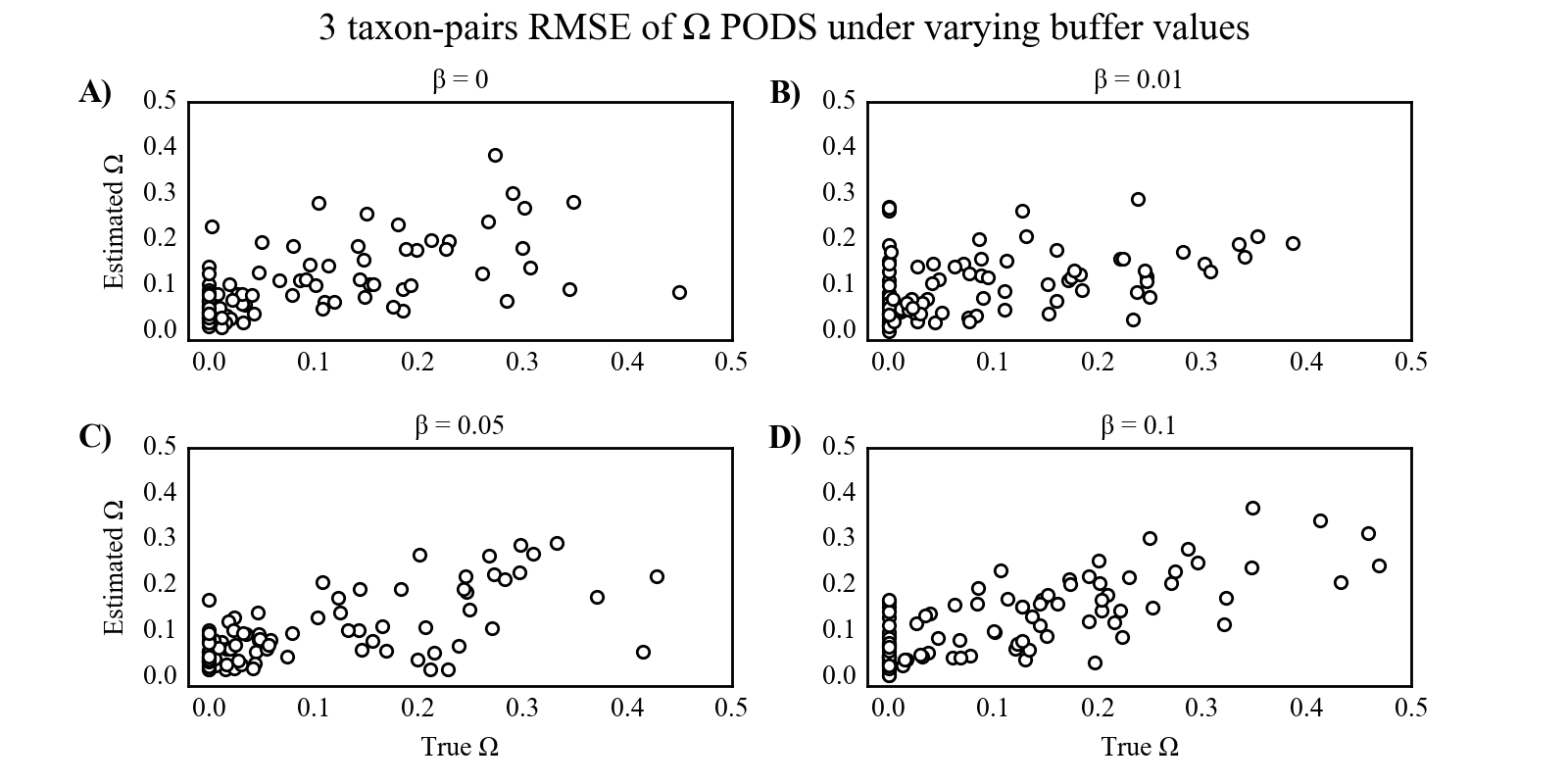


Scatterplots of RMSE in estimation of Ω as a function of true Ω for 100 PODS across different buffering regimes for the 3 taxon-pair data configuration. PODS were simulated and analyzed with a uniform prior on Ψ and sorted summary statistics using reference tables composed of 3 × 10^6^ samples from the prior. A) β = 0 B) β = 0.01 C) β = 0.05 D) β = 0.1

Figure S17


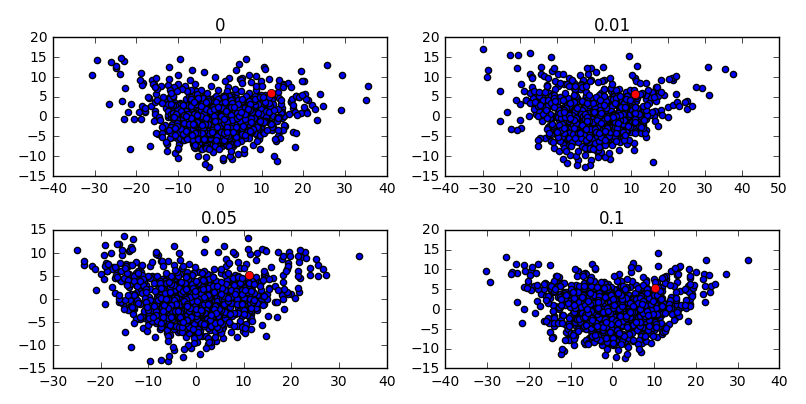


Plots of PC1 (x-axis) and PC2 (y-axis) for principal component analyses of the posterior predictive distribution and the observed data for the full Neotropical butterfly empirical analysis (116 taxon-pairs) under different buffering regimes. The red dot is the observed data, and the blue dots are posterior predictive simulations.

Figure S18


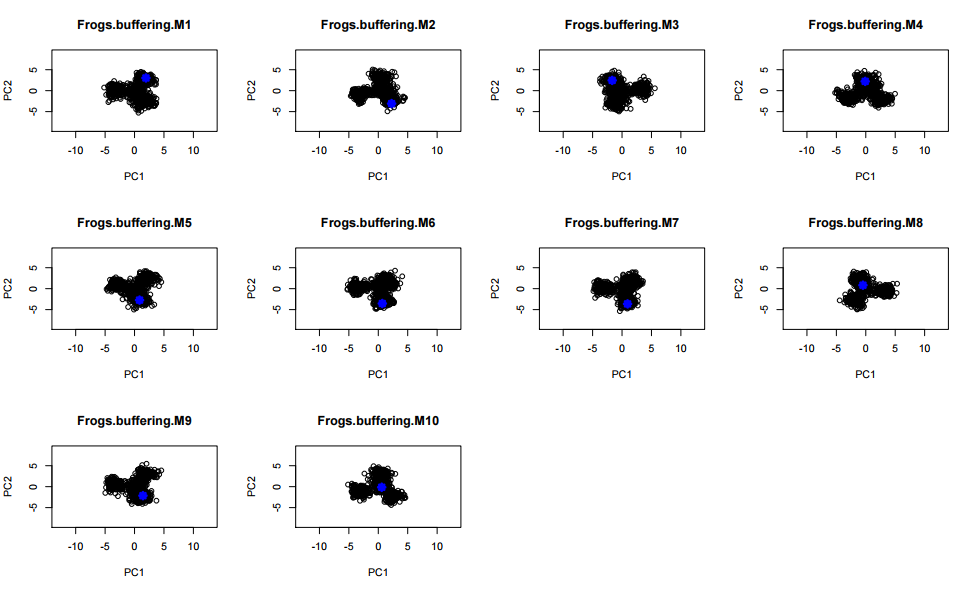


Plots of PC1 (x-axis) and PC2 (y-axis) for principal component analyses of the posterior predictive distribution and the observed data for the 4 taxon-pair Panamanian frogs empirical analysis under different buffering regimes. The blue dot is the observed data, and the black dots are posterior predictive simulations.

Figure S19


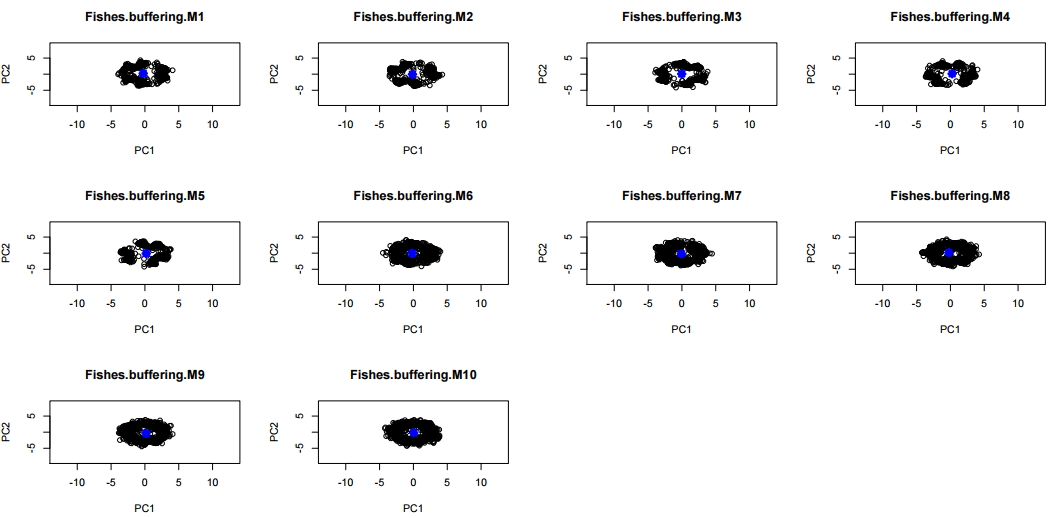


Plots of PC1 (x-axis) and PC2 (y-axis) for principal component analyses of the posterior predictive distribution and the observed data for the 3 taxon-pair Panamanian fishes empirical analysis under different buffering regimes. The blue dot is the observed data, and the black dots are posterior predictive simulations.

Figure S20


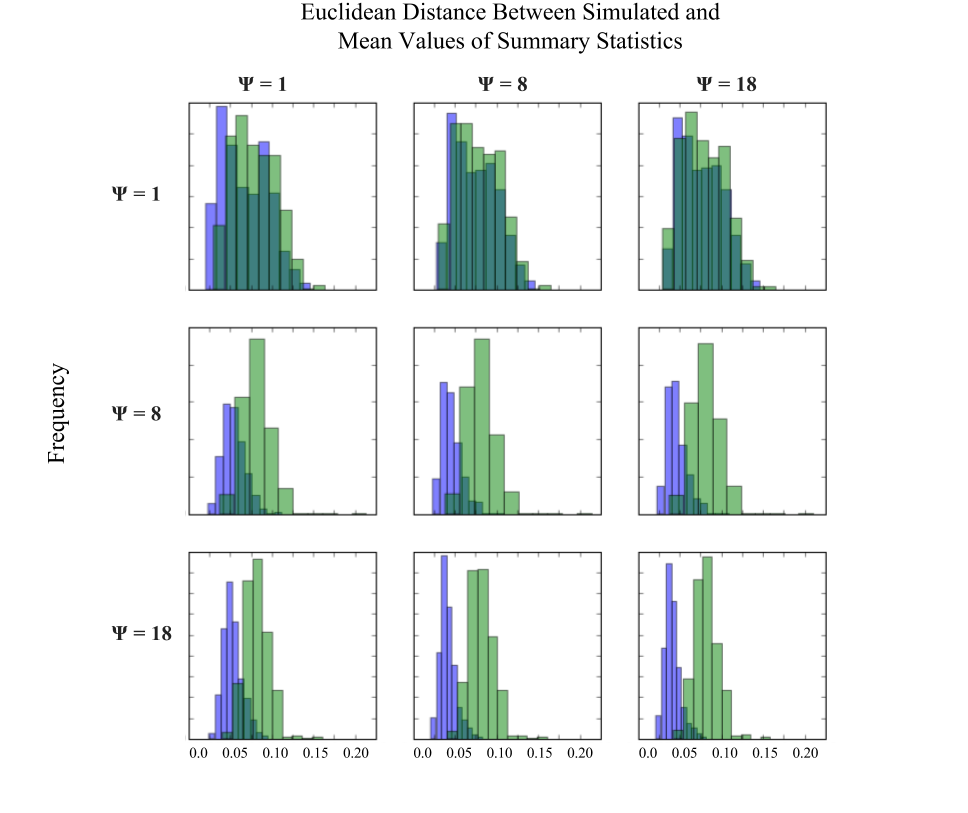


Plots of the Euclidean distance between simulated summary statistics and the average over all simulations per summary statistic class for different values of Ψ. Columns indicate the value of Ψ for which the mean of each summary statistic bin was calculated. Rows indicate the true Ψ for 10,000 simulations for which summary statistics were calculated and compared to the mean. Sorted summary statistics are shown in blue and unsorted summary statistics are shown in green.
